# Targeted potent antimicrobial and antitumor oxygen-heterocyclic-based pyran analogues: synthesis and computational studies

**DOI:** 10.1038/s41598-024-59193-2

**Published:** 2024-04-29

**Authors:** Ashraf H. F. Abd El-Wahab, Rita M. Borik, Al-Anood M. Al-Dies, Ahmed M. Fouda, Hany M. Mohamed, Raafat A. El-Eisawy, Mohamed H. Sharaf, Abdullah Y. A. Alzahrani, Ahmed A. Elhenawy, Ahmed M. El-Agrody

**Affiliations:** 1 Department of Chemistry, College of Science, Jazan University, B.O. Box 114, 45142 Jazan, Kingdom of Saudi Arabia; 2 Chemistry Department, Umm Al-Qura University, Al-Qunfudah University College, 21912 Al-Qunfudah, Saudi Arabia; 3 Chemistry Department, Faculty of Science, King Khalid University, 61413 Abha, Saudi Arabia; 4 Chemistry Department, Faculty of Science, Al-Azhar University, Nasr City, 11884 Cairo Egypt; 5 Department of Chemistry, Faculty of Science, Al-Baha University, 65528 Al-Baha, Saudi Arabia; 6 Department of Botany and Microbiology, Faculty of Science, Al-Azhar University, Cairo, 11884 Egypt; 7 Department of Chemistry, Faculty of Science and Arts, King Khalid University, Mohail Assir, Saudi Arabia; 8 Chemistry Department, Faculty of Science and Art, AlBaha University, 65731 Al Bahah, Saudi Arabia

**Keywords:** Biochemistry, Cancer

## Abstract

The process of creating a series of 3-amino-1-aryl-8-methoxy-1*H*-benzo[*f*]chromene-2-carbonitriles (**4a-q**) involved reacting 6-methoxynaphthalen-2-ol (**1**), the appropriate aromatic aldehydes (**2a-q**), and malononitrile (**3**) in an absolute ethanol/piperidine solution under Ultrasonic irradiation. However, the attempt to create 3-amino-1-aryl-1*H*-benzo[*f*]chromene-2,8-dicarbonitrile (**6a, d, e**) was unsuccessful when 6-cyanonaphthalen-2-ol (**5**) was stirred at room temperature, reflux, Microwave irradiation, or Ultrasonic irradiation. In addition, the target molecules were screened against *Staphylococcus aureus (MRSA)*, *Staphylococcus aureus*, *Bacillus subtilis, Bacillus cereus, Escherichia coli* and *Klebsiella pneumonia,* as well as a panel of three human cancer cells lines such as MCF-7, HCT-116, HepG-2 and two normal cell lines HFL-1 and WI-38. The obtained results confirmed that the pyran derivatives (**4 m, i, k**) which have a double chlorine at 3,4/2,3/2,5-positions, a single halogen atom 3-Cl/4-Br (**4c, e**) and a double bromine at 3,5-positions with a single methoxy group at 2-position (**4n**), of phenyl ring, and, to a lesser extent, other pyran derivatives with monoihalogenated (**4a, b, d, f**), dihalogenated (**4 g, h, j, l**) or trisubstituent phenyl ring (**4o, p, q**). Furthermore, compounds **4b-e, g, i, j, m,** and **n** showed negligible activity against the two normal cell lines, HFL-1 and WI-38. Moreover, compound **4 g** exhibited the strongest antimicrobial activity among the other pyran derivatives (**4a-f, g-q**) when compared to Ciprofloxacin. The MIC was assessed and screened for compound **4 g**, revealing bactericidal effects. Lastly, SAR and molecular docking were studied.

## Introduction

Cancer remains one of the leading causes of death and a major public health concern^1^. As a result, the creation of fresh strategies for the effective management of these illnesses has received increased attention. Because of their structural diversity, the nuclei of chromene and benzochromene have emerged as a promising and appealing scaffold in the development of antimicrobial and antitumor agents^2–31^. Specifically, a number of investigations have demonstrated the antimicrobial properties of benzochromene derivatives^2–6^.

**Figure 1:**
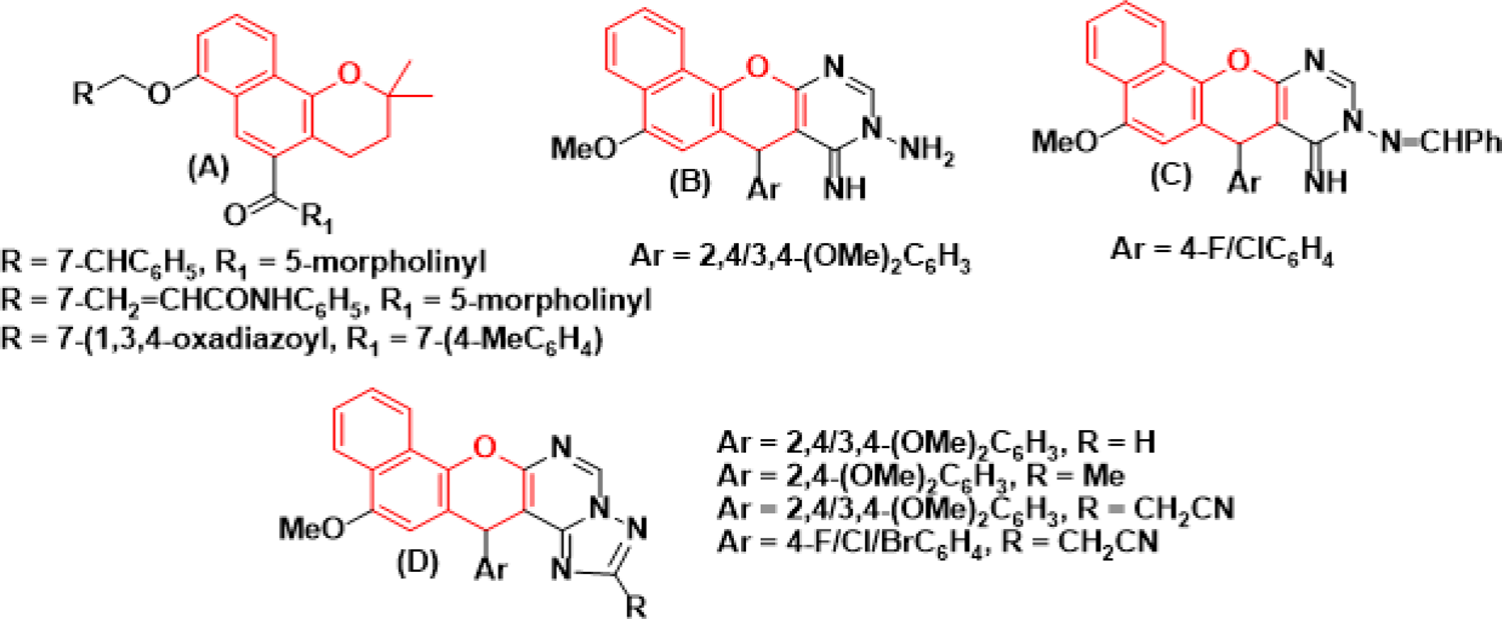
Structure of some benzo[*h*]chromene derivatives (red highlighted) with antimicrobial activities.

For instance, Fig. 1 illustrates how 2*H*-benzo[*h*]chromene derivatives (**A**) target AcrB and reverse bacterial multi drug resistance^2^, while 6-methoxy-4*H*-benzo[*h*]chromene derivatives (**B-D**) targeted strong antimicrobial activities and had an inhibitory effect against 14*α*-demethylase and DNA^3,4^. The 3-nitrile and 3-ester derivatives of 9-hydroxyof 1*H*-benzo[*f*]chromene **(E)** have a higher significant potent antibacterial and antifungal activities^5,6^.

**Figure 2:**
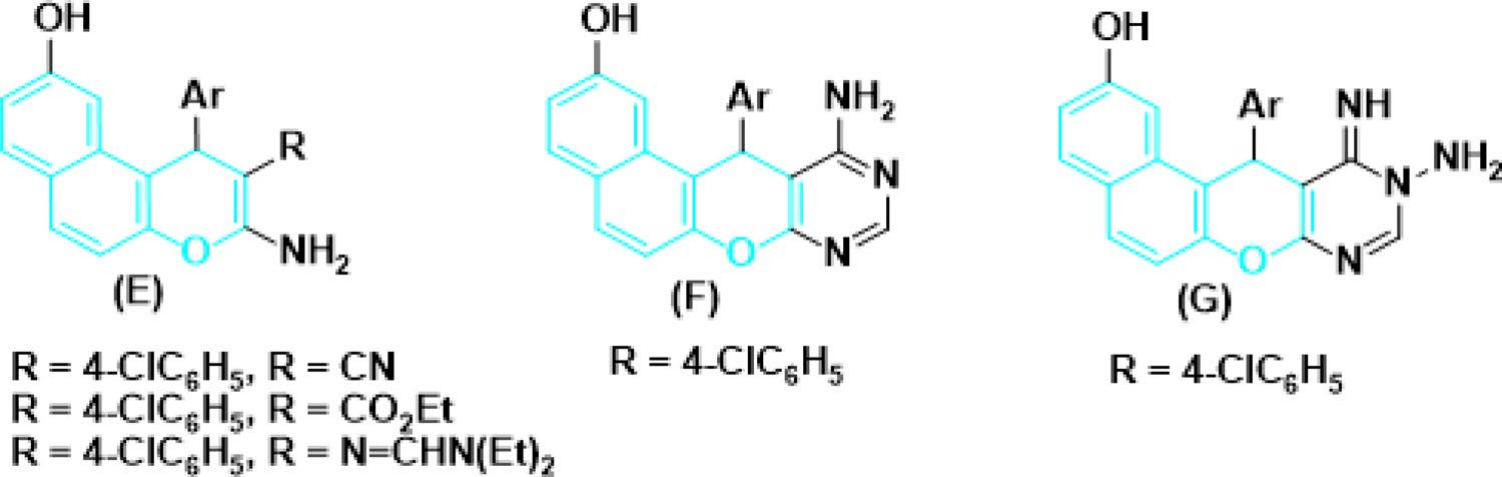
Structure of some benzo[*f*]chromene derivatives (light blue highlighted) with antimicrobial activities.

Figure 2 illustrates these additional findings. The 11-amino derivative of 9-hydroxy of 1*H*-benzo[*f*]chromene **(F)** exhibit good antimicrobial activities and the pyrimidino derivative of 9-hydroxyof 1*H*-benzo[*f*]chromene **(G)** acts as antimicrobial agents^6^.

**Figure 3:**
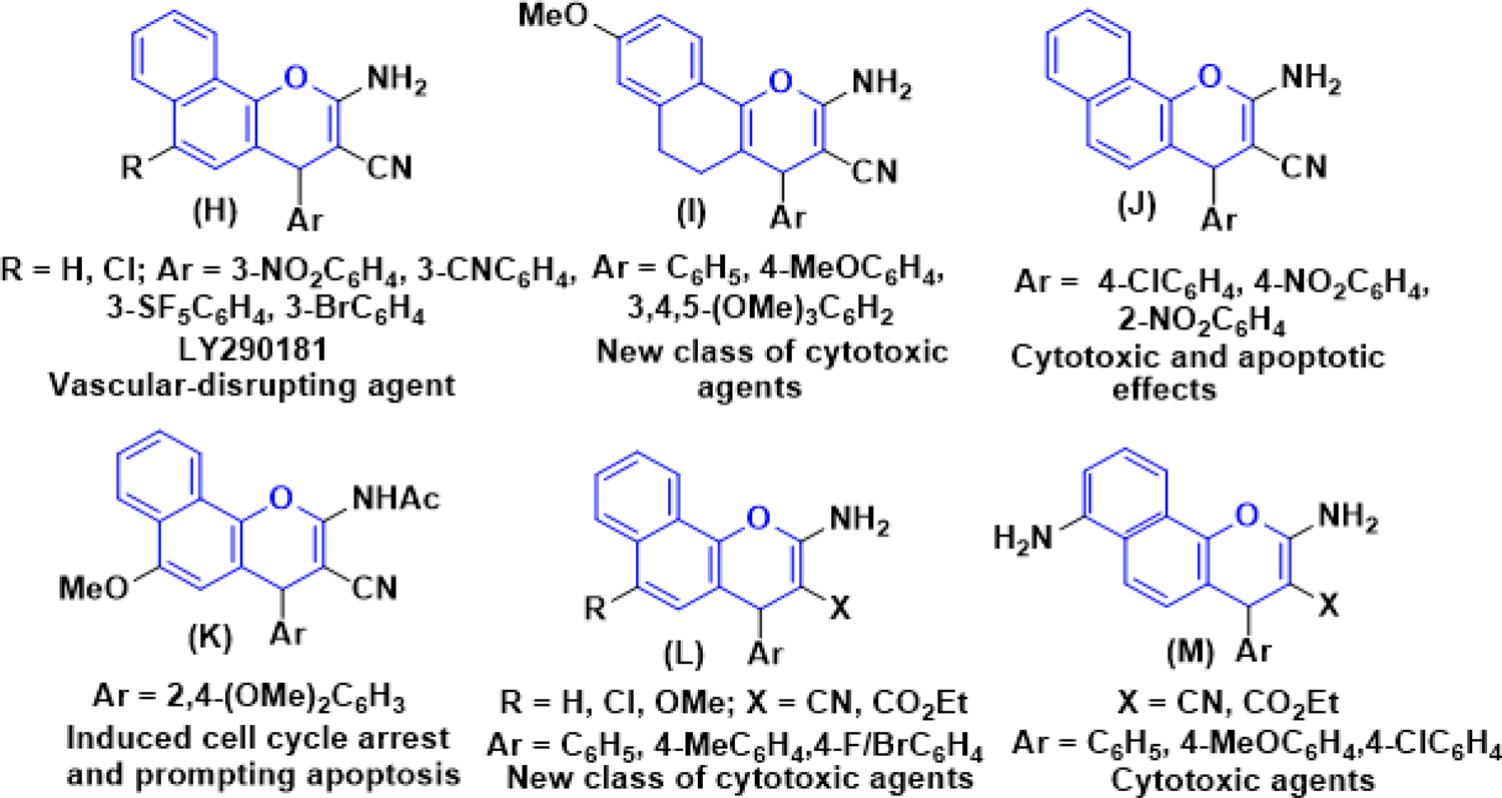
Structure of some 4*H*-benzo[*h*]chromene derivatives (Blue highlighted) with cytotoxic and apoptotic effects.

Derivatives of benzo[*h*]chromene are also a very effective option for treating a variety of human illnesses. For example, 4*H*-benzo[*h*]chromene analogues of LY290181 (**H**) acts as potential tumor vascular-disrupting agents^14^, 5,6-dihydro derivative of 4*H*-benzo[*h*]chromene (**I**) demonstrated a novel class of cytotoxic agents^15,16^, and 4-aryl derivatives of 4*H*-benzo[*h*]chromene (**J**) has cytotoxic and apoptotic effects on human cancer cell lines^17^, 2-acetylamino derivative of 6-methoxy-4*H*-benzo[*h*]chromene (**K**) induced cell cycle arrest and prompting apoptosis^18^, 3-carbonitrile/carboxylate derivatives of 4*H*-benzo[*h*]chromene^4^, 6-chloro/methoxy derivatives of 4*H*-benzo[*h*]chromene and 4*H*-benzo[*h*]chromene-3-carboxylate (**L**)^20–23^, 2,7-diamino derivatives of 4*H*-benzo[*h*]chromen-3-carbonitriles and ethyl 4*H*-benzo[*h*]chromene-3-carboxylates (**M**)^19^ have been reported as active cytotoxic agents against breast adenocarcinoma (MCF-7), human colon carcinoma (HCT-116) and hepatocellular carcinoma (HepG-2), respectively as shown in Fig. 3.

**Figure 4:**
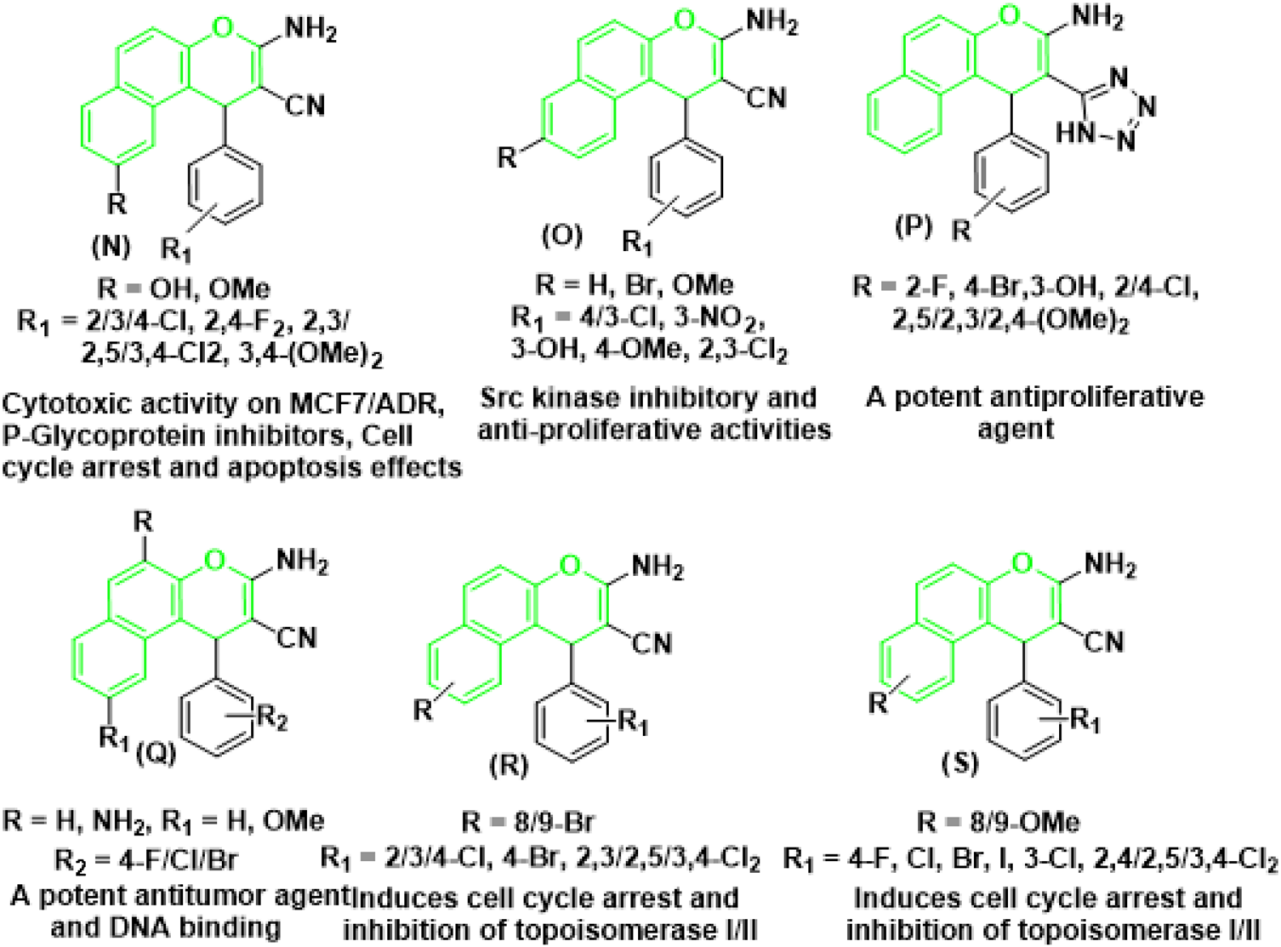
Structure of some 1*H*-benzo[*f*]chromene derivatives (green highlighted) with cytotoxic and apoptotic effects.

Similarly, 1*H*-benzo[*f*]chromene derivatives are regarded as promising lead candidates for anticancer drug development. For example, 9-hydroxy/methoxy of 1*H*-benzo[*f*]chromene derivatives (**N**) effective cytotoxic activity on MCF7/ADR, *P*-Glycoprotein inhibitors, Cell cycle arrest and apoptosis effects^7,8^, 1*H*-benzo[*f*]chromene derivatives and 8-bromo/methoxy derivative of 1*H*-benzo[*f*]chromenes **(O)** exhibited *c-Src* kinase inhibitory and proapoptotic activities^24,25^. A series of 1-substituted aryl-2-(1*H*-tetrazol-5-yl)-1*H*-benzo[*f*]chromene-3-amines **(P)** derivatives^27^, some derivatives of 3,5-diamino and 3-amino of 1*H*-benzo[*f*]chromene-2-carbonitrile **(Q)**, have been reported to exhibit cytotoxic and apoptotic effects against a variety of human cancer cell lines^17,28,29^. Aryl-substituted derivatives of 8/9-bromo **(R)**^31,32^ and 8/9-methoxy **(S)**^33,34^ of 1*H*-benzo[*f*]chromene have been shown to induce cell-cycle arrest and apoptosis in human cancer cells via dual inhibition of topoisomerases and tubulin as shown in Fig. 4.

**Figure 5:**
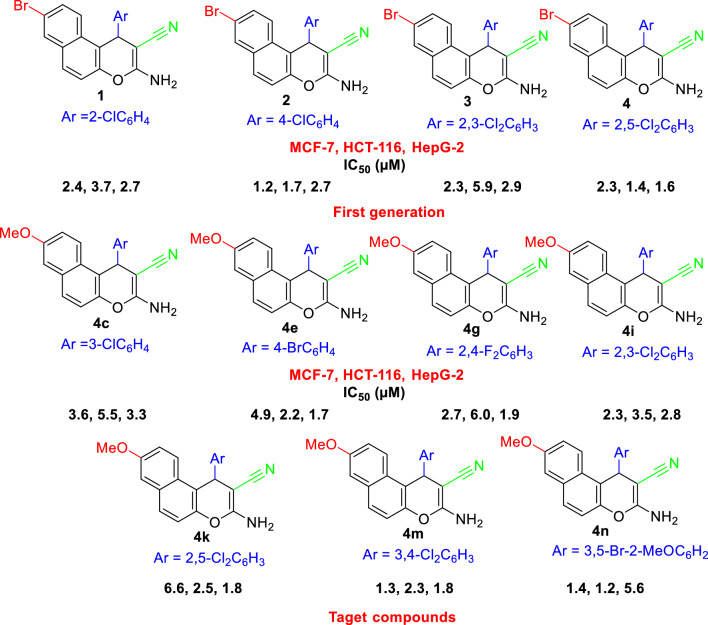
Designing strategy of 1*H*-benzo[*f*]chromene derivatives.

The designing strategy included the synthesis, antimicrobial and cytotoxic activities of the target compounds and comparative analyses of the result of cytotoxic activities regarding the performances of the freshly prepared molecules with a methoxy group at the 8-position and the formerly prepared molecules with a bromine atom at the 8-position^32^ as illustrated in in Fig. 5.

The final feature in this rationale study revealed that the recent molecules **4c**, **4e**, **4 g**, **4i**, **4 k**, **4 m,** and **4n** possessed a remarkable influence regarding their behaviors against tumor cells, which had an elevated potency in comparison with the molecules **1–4**^32^.

We report here the synthesis, antimicrobial and cytotoxic activities of 3-amino-1-aryl-8-methoxy-1*H*-benzo[*f*]chromene-2-carbonitrile derivatives, as a continuation of our ongoing research efforts for potent oxygen–nitrogen-heterocyclic-based with effective cytotoxic and antimicrobial activities^35–48^.

## Results and discussion

### Chemistry

**Figure 6:**
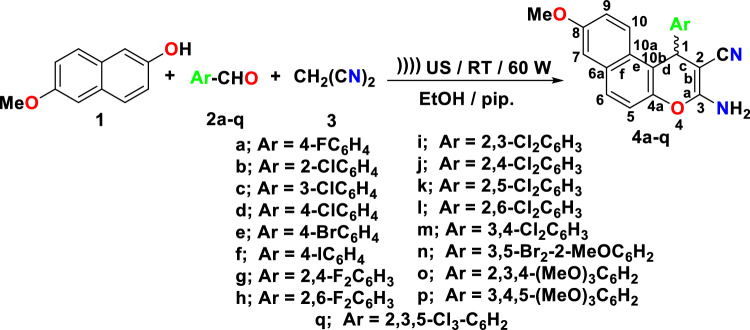
Synthesis of halogenated 1*H*-benzo[*f*]chromene derivatives (4a-q).

The synthesis of 3-amino-1-aryl-8-methoxy-1*H*-benzo[*f*]chromene-2-carbonitriles (**4a-q**) is illustrated in Fig. 6. Using a novel synthetic approach, Ultrasound irradiation, substituted phenyl at position 1 of *β*-enaminonitrile (**4a-q**) has been obtained. 6-Methoxynaphthalen-2-ol (**1**), the suitable aromatic aldehydes (**2a-q**), and malononitrile (**3**) interacted to form *β*-enamionitriles with 8-methoxy-1*H*-benzo[*f*]chromene motifs (**4a-q**) in an absolute ethanol/piperidine solution at room temperature and under 60 W Ultrasonic irradiation conditions as illustrated in Fig. 6.

**Figure 7:**
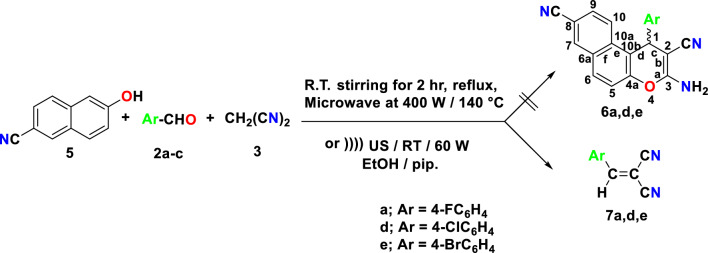
Attempted to synthesis of halogenated 8-cyano-1*H*-benzo[*f*]chromene derivatives (**6a, d, e**).

Furthermore, using stirred at room temperature, reflux conditions, Microwave irradiation conditions for two minutes at 140 °C, or 60 W of Ultrasonic irradiation conditions at room temperature, it was possible to achieve a one-pot, multicomponent reaction of 6-cyanonaphthalen-2-ol (**5**) with appropriate aromatic aldehydes (**2a, d, e**) and malononitrile (**3**) in an absolute ethanol/piperidine solution. This did not yield 3-amino-1-aryl-1*H*-benzo[*f*]chromene-2,8-dicarbonitrile (**6a, d, e**) as depicted in Fig. 7.

**Figure 8:**
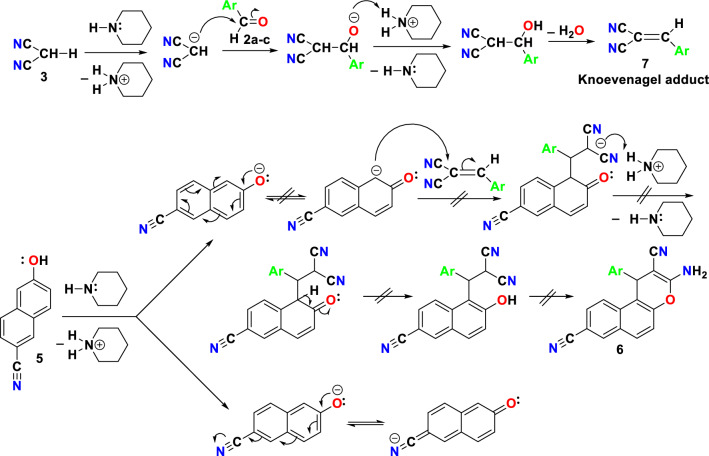
The unreactivity of 6-cyanonaphthalen-2-ol (5) towards Knoevenagel adduct (7a-c).

All of the cases showed that the 2-(4-halobenzylidene)malononitriles (**7a, d, e**) (Knoevenagel adducts) were separated, as shown by identical infrared spectra, and mixed m.p. The presence of the electron-withdrawing cyano group in 6-cyanonaphthalen-2-ol (**5**) may be the cause of its unreactivity towards 2-(4-halobenzylidene)malononitrile (**7a, d, e**) (Knoevenagel adducts). This property may alter the resonance within the ring as demonstrated in Fig. 8.

It's also critical to keep in mind that the 1-position in compound **4a-q**, is a chiral center^8^ and that each reaction was carefully regulated using the TLC method.

Spectral data verified the structure and purity of the synthesis compounds **4a-q**, and single crystal X-ray analysis of compounds **4 k, a, e, h, j,** and **n**^42,49–53^ offered a definitive confirmation for the desired molecules (refer to the Supplementary data).

### Biological activities

#### Antimicrobial assay

**Figure 9:**
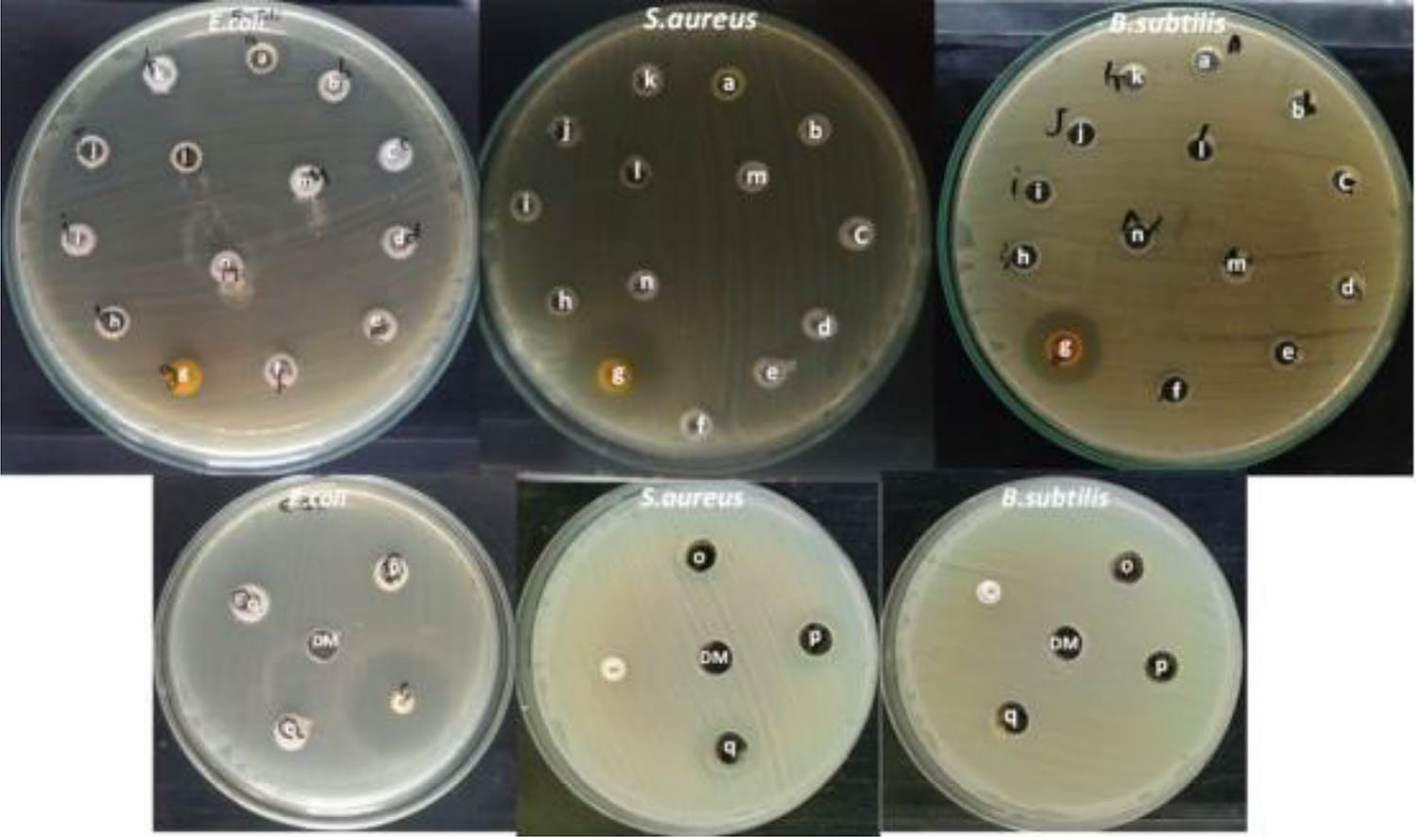
Antibacterial activity of compounds **4a-q** against different multi drug resistant pathogenic bacteria. *C* = Antibiotic (Ciprofloxacin), and *DM* = Negative control (DMSO).

The antibacterial activity of *β*-enamionitriles (**4a-q**) was screened using Mueller–Hinton agar medium for bacteria in an agar diffusion methodology^54^. Ciprofloxacin (1 mg/mL) was the reference antibiotic drug used in the analysis of collections that included four gram-positive species of multi drug resistant pathogenic bacteria: *Staphylococcus aureus (MRSA) (ATCC 6539)*, *Staphylococcus aureus (ATCC 6538), Bacillus cereus (ATCC 10987), Bacillus subtilis (ATCC 6633),* and two gram-negative species of multi drug resistant pathogenic bacteria *Klebsiella pneumonia (ATCC 13883)* and *Escherichia coli (ATCC 8739)*. The selection of these particular bacteria was stimulated by the affirmed antiproliferative activity of reported chromene and fused chromene derivatives^1–6,10,28^. Using a 1 mg/mL concentration of compounds (**4a-q**), the minimum zone of inhibition (IZ, the area around the antimicrobial agent where bacterial growth is prevented) was established in mm ± standard deviation beyond the well diameter (6 mm). When used as a blank, dimethyl sulfoxide (DMSO) showed no antimicrobial activity. The synthetic compound's inhibitory effects on evaluation against these organisms are depicted in Fig. 9 and Table 1.

**Table 1:** Antibacterial screening for compounds (**4a-q**).

Diameter of inhibition zone (mm)						
Cpd	*Gram* + *ve bacteria*			*Gram –ve bacteria*		
	S. *Aureus (MRSA)*	*S. aureus*	*B. subtilis*	*B. cereus*	*E. coli*	*K. pneumonia*
4a	14	0	0	13	11	14
4b	13	0	0	14	12	13
4c	15	0	0	17	12	13
4d	12	0	0	15	0	12
4e	12	0	0	14	0	10
4f	12	0	8	15	0	9
4g	17	23	21	20	14	16
4h	13	9	0	9	0	10
4i	13	0	0	8	0	14
4j	13	0	0	8	12	14
4k	12	0	0	0	13	15
4l	13	0	0	8	0	14
4m	14	0	0	13	10	13
4n	14	0	0	13	0	11
4o	12	10	0	14	12	11
4p	13	11	0	15	11	12
4q	13	10	10	13	12	13
Ciprofloxacin	0	0	0	22	25	0
DMSO	0	0	0	0	0	0

Compound **4 g** is more effective than other pyran derivatives (**4a-f, g-q**) against bacteria that are resistant to multiple drugs, as demonstrated by Table 1.

#### Minimum inhibitory concentration (MIC)

**Figure 10:**
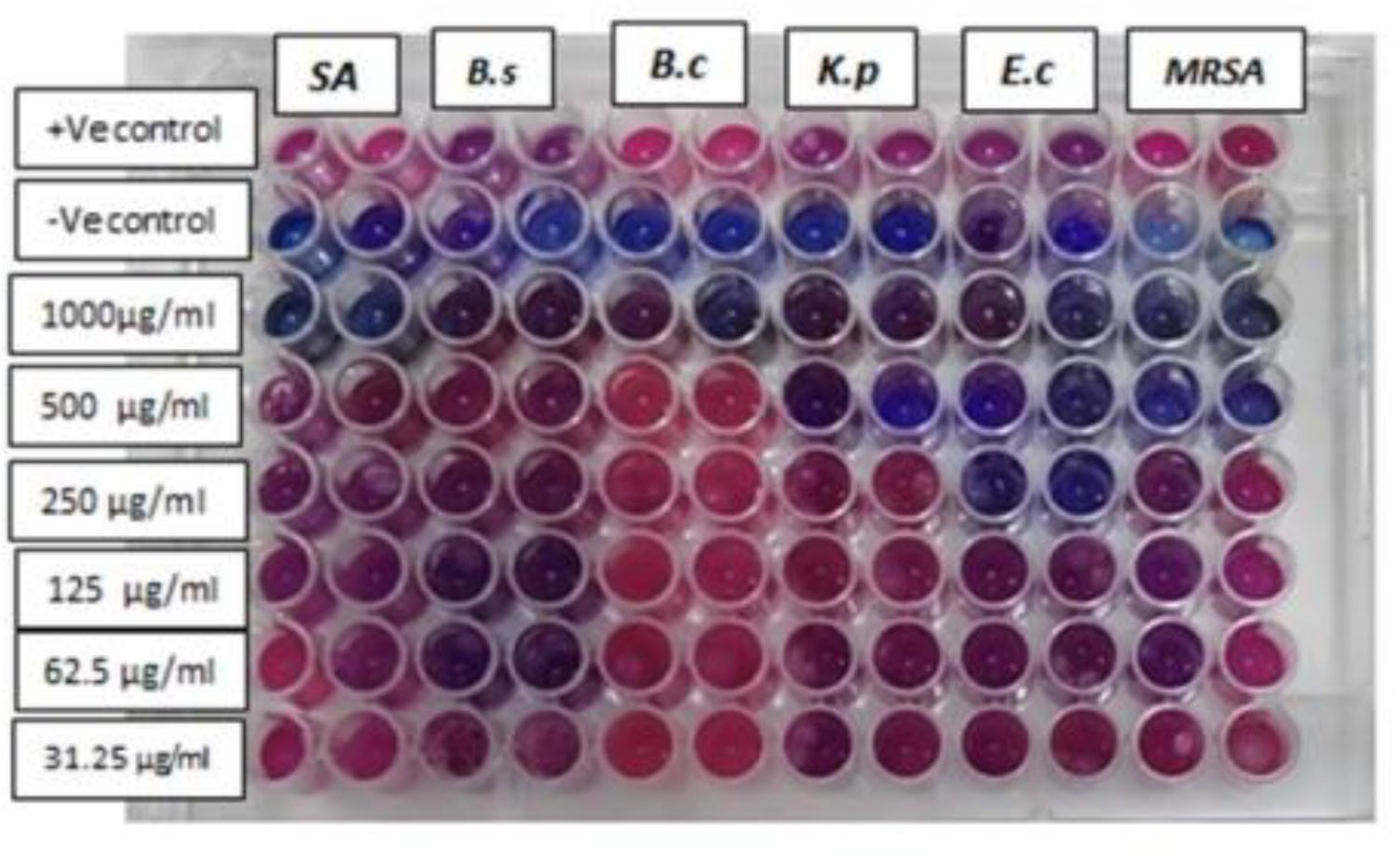
MIC of compound **4 g** on *Staphylococcus aureus* (MRSA)*, Staphylococcus aureus, Bacillus subtilis, Bacillus cereus, Escherichia coli* and *Klebsiella pneumonia* plates after 24 h in Mueller Hinton (MH) broth resazurin assay [pink colour indicates growth and blue means inhibition of growth; the test organism, Positive Control (MH broth + bacterial suspension + indicator) without compound; Negative or sterility control (MH broth + sterile distilled water + indicator) without bacteria.

The minimum inhibitory concentration, or MIC, is the lowest concentration needed to stop bacterial growth. When evaluating how well antimicrobial agents work against various bacterial strains, MIC values are crucial. The antimicrobial agent is more effective against the bacterial strain the lower the MIC value. The right dosage of the antimicrobial agent to be used in the treatment of bacterial infections can also be determined with the aid of the MIC value^55^. Table 2 and Fig. 10 show the minimum inhibitory concentration (MIC) of the active compound **4 g** against various bacterial strains.

**Table 2:** MIC of compound **4 g** against multi drug resistant bacterial strains.

Microbial strain	(MIC) of compound 4 g against bacterial strains (µg/ml)
*Staphylococcus aureus (MRSA)*	500
*Staphylococcus aureus*	1000
*Bacillus subtilis*	1000
*Bacillus cereus*	1000
*Escherichia coli*	250
*Klebsiella pneumonia*	500

The Table 2 shows the minimum inhibitory concentration (MIC) of the active compound **4 g** against different bacterial strains. The minimum inhibitory concentration (MIC) of an antimicrobial agent is the concentration at which the microorganism cannot grow visible. According to Table 2, the active compound **4 g** has a minimum inhibitory concentration (MIC) of 1000 µg/ml against *Bacillus cereus*, *Bacillus subtilis*, and *Staphylococcus aureus*. However, its MIC for *Staphylococcus aureus (MRSA)* and *Klebsiella pneumonia* is 500 µg/ml. When it comes to *Escherichia coli*, the lowest MIC value is 250.

#### Antitumor assay

**Figure 11:**
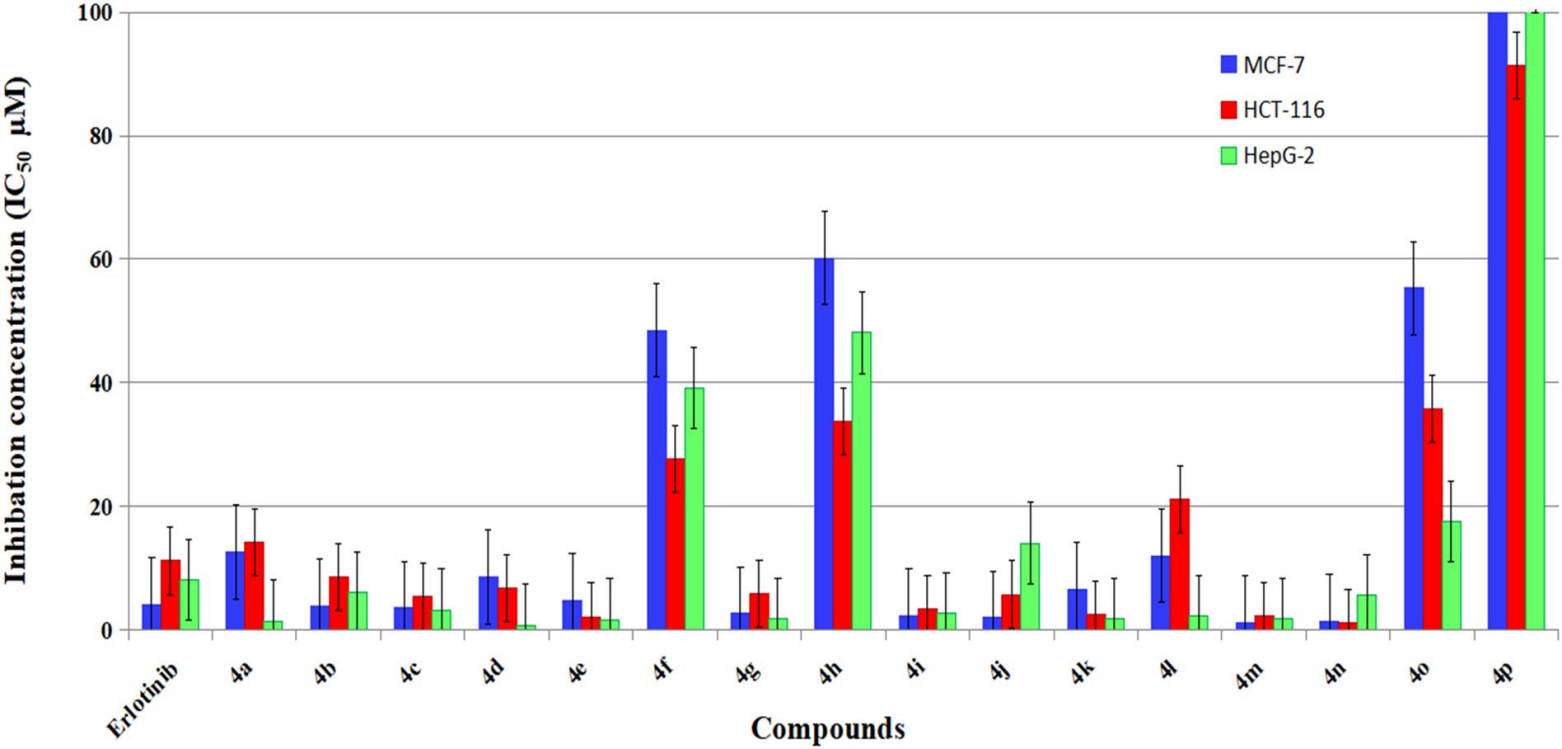
IC_50_ values expressed in (µM) of halogenated 1*H*-benzo[*f*]chromene derivatives 4a-q against MCF-7, HCT and HepG-2 tumor cells.

The antiproliferative activity of the newly synthesized 1*H*-benzo[*f*]chromenes derivatives (**4a-q**) was examined in three human cancer cell lines: MCF-7 (breast cancer), HCT-116 (human colon cancer), and HepG-2 (hepatocellular carcinoma), as well as the two normal cell lines, HFL-1 (human foetal lung) and WI-38 (human diploid fibroblasts), using the 3-(4,5-dimethylthiazol-2-yl)-2,5-diphenyl tetrazolium bromide (MTT) colorimetric assay^56^. The selection of these particular cell lines, MCF-7, HCT-116 and HepG-2 was stimulated by the affirmed antiproliferative activity of reported chromene and fused chromene derivatives^7–34^. The three cell lines used in the experiments were exposed to reference cytotoxic compound, Erlotinib. The data were expressed as growth inhibitory concentration (IC_50_) values, Table 3 and Fig. 11. These values represent the concentrations of the compounds required to cause a 50% inhibition of cell growth after a 24-h incubation period relative to the untreated controls.

**Table 3:** Cytotoxic activity of target compounds against MCF-7, HCT-116 and HepG-2 cell lines.


Compound	R	IC_50_ (µM)^a^				
		Cancerotoxicity			Normotoxicity	
		MCF-7	HCT-116	HepG-2	HFL-1	WI-38
4a	4-F	12.7 ± 0.05^b^	14.2 ± 0.01^b^	1.5 ± 0.01^b^	–	–
4b	2-Cl	3.9 ± 0.18	8.6 ± 0.25	6.1 ± 0.2	198.6 ± 0.1	171.0 ± 0.2
4c	3-Cl	3.6 ± 0.14	5.5 ± 0.35	3.3 ± 0.5	187.6 ± 0.2	214.1 ± 0.1
4d	4-Cl	8.6 ± 0.29^b^	6.9 ± 0.97^b^	0.8 ± 0.08^b^	164.4 ± 1.1	165.5 ± 1.3
4e	4-Br	4.9 ± 0.25^b^	2.2 ± 0.58^b^	1.7 ± 0.13^b^	189.9 ± 1.1	165.3 ± 1.2
4f	4-I	48.5 ± 0.1	27.8 ± 0.3	39.2 ± 0.2	–	-
4g	2,4-F_2_	2.7 ± 0.23	6.0 ± 0.11	1.9 ± 0.5	179.4 ± 0.1	190.1 ± 0.3
4h	2,6-F_2_	60.2 ± 0.3	33.8 ± 0.12	48.1 ± 0.2	–	–
4i	2,3-Cl_2_	2.3 ± 0.01	3.5 ± 0.15	2.8 ± 0.17	154.5 ± 0.1	160.1 ± 0.2
4j	2,4-Cl_2_	2.0 ± 0.02	5.8 ± 0.16	14.1 ± 0.18	180.8 ± 0.3	177.8 ± 0.3
4k	2,5-Cl_2_	6.6 ± 0.4	2.5 ± 0.23	1.8 ± 0.03	–	–
4l	2,6-Cl_2_	12.1 ± 0.06	21.3 ± 0.01	2.3 ± 0.02	–	–
4m	3,4-Cl_2_	1.3 ± 0.21	2.3 ± 0.97	1.8 ± 0.06	181.0 ± 0.1	175.5 ± 0.2
4n	3,5-Br_2_-2-OMe	1.4 ± 0.05	1.2 ± 0.06	5.6 ± 0.21	139.5 ± 0.1	135.4 ± 0.3
4o	2,3,4-(OMe)	55.3 ± 0.01	35.9 ± 0.02	17.6 ± 0.05	–	–
4p	3,4,5-(OMe)	114.4 ± 0.03	91.4 ± 0.01	106.5 ± 0.1	–	–
4q	2,3,5-Cl_3_	110.4 ± 0.1	107.2 ± 1.6	76.5 ± 0.9	–	–
Erlotinib	–	4.16 ± 0.2	11.21 ± 0.6	8.19 ± 0.4	14.0 ± 1.2	28.5 ± 0.2

^a^IC_50_ values expressed in **µM** as the mean values of triplicate wells from at least three experiments and are reported as the mean ± standard error and^b^ ^25^.

The majority of the prepared compounds demonstrated excellent growth inhibitory activity against the tested cancer cell lines, as explained by the results shown in Table 1. Compounds **4 m, n, j, i, g, c,** and **b** were emerged as the most potent counterpart against MCF-7 in this study; their IC_50_ values ranged from 1.3 to 3.87 µM. These compounds were found to be 3.2, 3.0, 2.1, 1.8, 1.5, 1.2, and 1.1 times more active than Erlotinib (4.16 ± 0.2 µM). Meanwhile, compound **4e** (IC_50_ = 4.9 µM) was almost equipotent as Erlotinib (4.16 ± 0.2 µM). The compounds **4n, e, m, k, i, c, j, g, d,** and **b** (IC_50_ = 1.2–8.6 µM) were also found to be more potent and effective than Erlotinib (IC_50_ = 11.21 µM). These findings were obtained from cytotoxicity evaluation in the HCT-116 cell line. In terms of their ability to inhibit HepG-2, compounds **4d, a, e, k, m, g, l, i, c, n,** and **b** showed strong potency (IC_50_ = 0.8–6.1 µM) against HepG-2 compared to Erlotinib (IC_50_ = 8.19) Compounds **4b-e, g, i, m,** and **n** additionally showed a weak growth inhibitory effect on the normal cell lines HFL-1 and WI-38, with IC_50_ values ranging from 80.8 to 214.1 µM. In comparison to Erlotinib, the remaining compounds demonstrated equipotent or moderate to fair cytotoxic activities against the three types of tumour cells.

#### SAR studies

Several essential structural requirements were identified by the SAR study, which improved the potency of these 1*H*-benzo[*f*]chromene derivatives (**4a-q**). The difference in the substitution pattern, including the type of substituent (electron-donating groups, electron-withdrawing groups, or both of them) and their position on the phenyl ring at the 1-position of 1*H*-benzo[*f*]chromene moiety, affected the cytotoxic activity of the synthesized benzochromene derivatives toward the tested cancer cell lines. The observed order of improved potency against MCF-7 with different substituents on the phenyl ring at 1-position of 1*H*-benzo[*f*]chromene moiety could be represented as 3,4-Cl_2_ > 3,5-Br_2_-2-OMe > 2,4-Cl_2_ > 2,3-Cl_2_ > 2,4-F_2_ > 3-Cl > 2-Cl > 4-Br > 2,5-Cl_2_ > 4-Cl > 4-F > 2,6-Cl_2_ > 2,6-F_2_ > 4-I > 2,3,4-(OMe)_3_ > 2,3,5-Cl_3_ > 3,4,5-(OMe)_3_. These results are intimating that the grafting of a lipophilic electron-withdrawing (disubstituted, chlorine atoms at 3,4-, 2,4-, 2,3-, 2,5-position or fluorine atoms at 2,4-position), a lipophilic electron-withdrawing together with a lipophilic electron donating (trisubstituted, bromine atoms at 3,5-position and methoxy group at 2-position) and a lipophilic electron-withdrawing (monosubstituted chlorine atom at 3-, 2-position, bromine atom at 4-position) is more beneficial than the other lipophilic electron-withdrawing substituent on the phenyl ring at 1-position of 1*H*-benzo[*f*]chromene moiety with a lipophilic electron donating methoxy group at 8-position and the disubstituted is more active than trisubstituted and monosubstituted.

The order of anticancer activities of the *β*-enaminonitrile derivatives (**4a-q)** against HCT-116 has widely varied in accordance to the position and the type of the substituent on the phenyl ring at 1-position of 1*H*-benzo[*f*]chromene moiety, the activities were decreased in the order of 3,5-Br_2_-2-OMe > 4-Br > 3,4-Cl_2_ > 2,5-Cl_2_ > 2,3-Cl_2_ > 3-Cl > 2,4-F_2_ > 2,4-Cl_2_ > 4-Cl > 2-Cl > 4-F > 2,6-Cl_2_ > 2,6-F_2_ > 4-I > 2,3,4-(OMe)_3_ > 3,4,5-(OMe)_3_ > 2,3,5-Cl_3_, suggesting that the position and the type of the bulky substituent incorporation may be advantageous.

Regarding the activity against HepG-2, the order of the antitumor activity of the 1*H*-benzo[*f*]chromene derivatives **(4a-q)** was found to be 4-Cl > 4-F > 4-Br > 2,4-F_2_ > 2,5-Cl_2_ > 3,4-Cl_2_ > 2,6-Cl_2_ > 2,3-Cl_2_ > 3-Cl > 2-Cl > 3,5-Br_2_-2-OMe > 2,4-Cl_2_ > 2,3,4-(OMe)_3_ > 2,3,5-Cl_3_ > 2,6-F_2_ > 4-I > 3,4,5-(OMe)_3_. These results suggested that the monsubstituted phenyl (lipophilic electron-withdrawing group) is preferred as an antitumor agent in comparison to the disubstituted phenyl (lipophilic electron-withdrawing group) and trisubstituted phenyl (lipophilic electron-withdrawing together with a lipophilic electron donating and a lipophilic electron donating). Furthermore, compounds **4b-e, g, i, j, m,** and** n** have been screened against two normal cell lines, HFL-1 and WI-38 and displayed IC_50_ ranging from 80.8 to 214.1 µM, which confirm their inadequate performance against these control cell lines. In addition, compound **4 g** with 2,4-F_2_ at the phenyl group at the 1-position of the 1*H*-benzo[*f*]chromene moiety exhibited the highest antimicrobial activity than the other pyran derivatives (**4a-f, g-q**).

Finally, we can deduce that the position and the type of the substituent on the phenyl group at the 1-position of the 1*H*-benzo[*f*]chromene moiety played a vital role in its antitumor activity with a lipophilic electron donating methoxy group at 8-position.

#### Molecular docking

The docking analysis was performed to explicate the potency of these desirable molecules in vitro against the kinase through their potential interaction mechanisms with their crystal frameworks “DHFR” dihydrofolate reductase [PDB : 3FY8^57^] and “EGFR” tyrosine kinase PDB : 4HJO^58^].

2*H*-Chromen-2-one derivatives have shown potential as DHFR inhibitors due to their structural resemblance to folate. By docking 2*H*-chromen-2-one into the DHFR active site, we can explore potential binding interactions and evaluate its inhibitory activity. EGFR is over expressed in various cancers, making it an attractive target for anticancer drug development. 2*H*-Chromen-2-one derivatives have demonstrated anti-EGFR activity by inhibiting EGFR auto-phosphorylation. Docking 2*H*-chromen-2-one into the ATP-binding site of EGFR allows us to explore potential interactions and assess its inhibitory effects. In summary, both DHFR and EGFR play critical roles in cellular processes, and their structures provide valuable information for rational drug design. By studying coumarin interactions with these proteins, we aim to uncover potential therapeutic applications^59,60^.

The docking investigation was implemented through Glide’s module.® The preliminary inhibitors 5-[[(2*R*)-2-cyclopropyl-7,8-dimethoxy-2*H*-chromen-5-yl]methyl]pyrimidine-2,4-diamine ”Erlotinib, AR-101″ for DHFR and *N*-(3-ethynylphenyl)-6,7-bis(2-methoxyethoxy)-4-quinazolinamine “Erlotinib” for EGFR were redocked into the peroxidase crystal framework to verify the docking methodology. Furthermore, the efficacious performance of the targeted molecules was authenticated via the low values of RMSD (2.01 Å) for DHFR and (2.75 Å) for EGFR, which were acquired through the root mean square deviation between the native and redocked poses of the co-crystallized inhibitor. We generated the 3D loop of DHFR using mGen-THERADER and used it in the docking framework. This loop contains seven amino acid residues, which are important for maintaining the conformation of the enzyme. Moreover, it is believed that the DHFR active site is located within this loop, making it an ideal target for docking studies. We used binding-energy BE to study the most active compounds with the DHFR receptor (**4 g**) and EGFR (**4i, 4 m** and **4n**), then compared them with the reference inhibitors “AR-101 and Erlotinib. The initial inhibitors have been adequately installed into their binding sites in order to attain their crystal configurations. The lowest score poses and RMSD revealed increased stability in the binding pocket. These data was utilized to rank the docked poses and to select the most capable docked conformation of each compound. We selected the most perforable docking conformations of all active compounds that were detected inside the active site with proper alignment. The DHFR active site including the hydrophilic amino acids (LYS32, ARG57 and TYR98). Where binding site for EGFR including hydrophobic (GLY772, MET769, LEU694) and hydrophilic (CYS773,LYS692) other which interact with the ligands, that are necessary for the enzyme's catalytic activity. We defined the inhibitory behaviour in the term of binding energy BE for all compounds **4a-q** that were evaluated with the receptor Table 4.

**Table 4:** The binding affinity (kcal/mol) of 4a–q hybrids against DHFR and EGFR receptors.

Cpd	ΔG	RMSD	H.B	Int	E_ele	Ki	Cpd	ΔG	RMSD	H.B	Int	E_ele	Ki
3FY8							4HJO						
4a	− 6.63	1.54	− 22.18	− 24.42	− 10.24	0.33	4a	− 6.37	1.99	− 26.36	− 16.63	− 10.55	0.90
4b	− 7.02	1.11	− 17.59	− 28.30	− 11.08	0.33	4b	− 7.17	1.10	− 21.50	− 20.83	− 9.86	0.89
4c	− 6.66	1.12	− 24.55	− 28.75	− 10.27	0.33	4c	− 6.90	2.56	− 16.48	− 24.06	− 10.03	0.89
4d	− 6.90	4.05	− 27.68	− 16.89	− 10.14	0.33	4d	− 6.42	1.01	− 15.23	− 24.97	− 10.21	0.90
4e	− 7.00	2.31	− 27.36	− 25.53	− 10.89	0.33	4e	− 6.80	1.12	− 12.76	− 19.29	− 10.43	0.89
4f	− 7.11	1.50	− 26.22	− 27.33	− 9.88	0.33	4f.	− 6.77	1.91	− 13.23	− 20.30	− 11.86	0.89
4g	− 6.83	1.49	− 31.98	− 20.48	− 10.02	0.33	4g	− 6.96	1.20	− 20.29	− 22.49	− 10.33	0.89
4h	− 6.58	0.78	− 40.50	− 24.06	− 9.93	0.33	4h	− 6.52	1.67	− 34.69	− 19.44	− 10.68	0.90
4i	− 6.81	1.62	− 22.24	− 26.39	− 9.27	0.33	4i	− 6.61	1.15	− 9.40	− 18.86	− 9.41	0.89
4j	− 7.14	1.56	− 23.30	− 21.37	− 9.60	0.33	4j	− 6.46	1.92	− 15.67	− 18.16	− 9.95	0.90
4k	− 6.53	2.93	− 39.71	− 18.26	− 8.83	0.33	4k	− 6.13	1.54	− 26.79	− 23.73	− 9.55	0.90
4l	− 6.94	1.65	− 18.26	− 23.55	− 10.15	0.33	4l	− 6.60	1.55	− 7.13	− 28.21	− 10.48	0.89
4m	− 7.47	2.37	− 25.77	− 27.70	− 10.44	0.33	4m	− 6.66	1.21	− 28.42	− 17.68	− 9.58	0.89
4n	− 8.14	1.52	5.35	− 20.62	− 9.14	0.33	4n	− 6.91	1.25	− 1.59	− 18.25	− 9.47	0.89
4o	− 8.03	2.26	8.12	− 28.17	− 9.44	0.33	4o	− 7.33	1.15	3.67	− 26.42	− 10.31	0.88
4p	− 6.98	1.45	− 29.10	− 22.63	− 9.85	0.33	4p	− 7.18	1.15	− 10.35	− 19.95	− 9.71	0.89
4q	− 7.52	1.81	− 14.97	− 23.09	− 9.33	0.33	4q	− 6.87	1.12	− 8.23	− 21.94	− 9.75	0.89
AR − 101	− 9.74	2.58	− 622.70	− 10.69	− 9.94	0.33	Erlotinib	− 5.36	1.34	− 8.17	− 17.97	− 9.20	0.91

Where *ΔG* free binding energy of the ligand, *RMSD* root-mean-square deviation, *H.B*. H-bonding energy between protein and ligand, *EInt* binding affinity of H-bond interaction with receptor, *Eele* electrostatic interaction over the receptor, and inhibition constant in µM.

**Figure 12:**
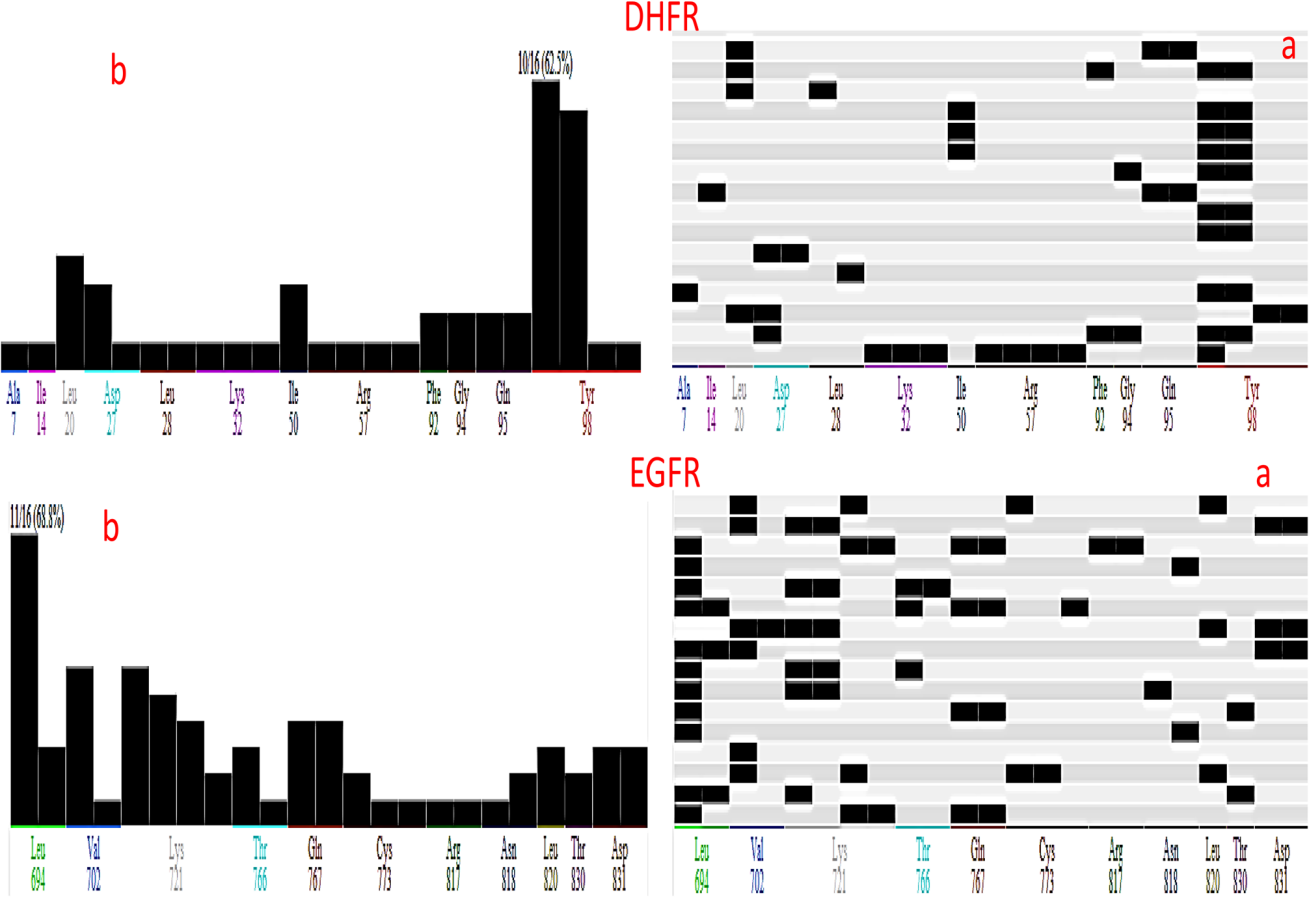
Validation docking analysis based on PLIF between **4a-q** and the DHFR and EGFR receptors [(**a**) barcodes, (**b**) populations].

Then redocked investigated compounds, compared the results to the reference inhibitors, and obtained a root mean square deviation (RMSD) using the interaction between reference inhibitors and protein. The " CHARMM " molecular-mechanics force field created the poses, then picked the Pose with the lowest "ΔG" and "RMSD" to evaluate the binding affinities of investgted molecules. The PLIF was computed based on the docking results of the inhibitors at the active sites of 3FY8 and 4HJO, which are the target proteins for the docking protocol validation. The binding effectiveness of the inhibitors **4a-q** was evaluated by the protein–ligand interaction fingerprint (PLIF) method, which is shown in Fig. 12.

The active site of EGFR interacts most strongly with LYS694, which has a 68.8% interaction score, while the active site of DHFR interacts most strongly with ASP98, which has a 62.5% interaction score. We classified the different types of interactions, such as ionic, hydrogen bonding, and surface interactions, according to the residues involved, and created a fingerprint scheme that serves as a database of the complex. All the docking conformations was submitted to PLIF which generate a population plot (Fig. 12).

For additional molecular docking experiment validation, inhibition constant Ki was estimated for investigated **4a-q** molecules. The Glide ΔG score, which calculates the free energy of binding between the ligand and the receptor protein, was used to assess the binding mechanism and stability of the docked investigated compounds.

#### In case 3FY8

**Figure 13:**
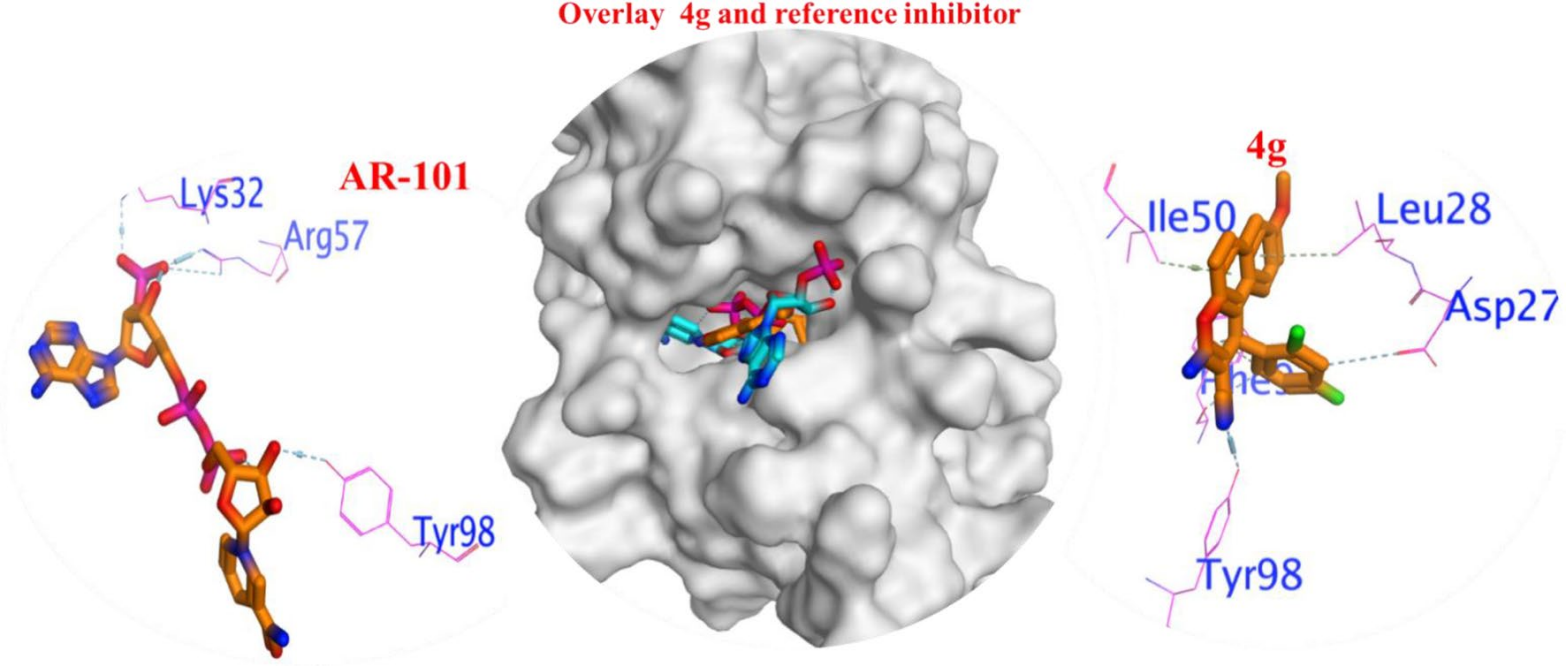
Binding interaction of most active compound **4 g** and reference inhibitor and superimposed in active site to validate the docking protocol.

The poses **4 g** showed lower binding efficiency (ΔG =  − 6.8 kcal/mol) with RMSD = 1.5 Å than AR-101 (ΔG =  − 9.7 kcal/mol) with RMSD = 2.6 Å, as listed in Table 4. The interactions between the all compounds and residues of active site were mainly polar bonds, hydrogen bonding, π − π, and π − H interactions, which contributed to a strong alignment with the enzyme backbone which contributed to a strong alignment with the enzyme backbone (Fig. 13).

All compounds demonstrated binding affinities rang (ΔG = − 6.4 to − 8.6 kcal/mol). The most active compound **4 g** comforting in the binding pocket by interacting by strong H-bond with the hydrophilic binding pocket ASP27 and TYR98, and hydrophobic residues LEU50 and Ile 28 (Fig. 13). It is inferred that the formation of strong interactions with important residues can pinpoint the EGFR binding pocket. Lastly, according to the 3D-molecular docking, the superiorly-active **4 g** and AR-101 prefer a parallel orientation between the central chromene ring and the important hydrophilic TYR98.

#### In case 4HJO

**Figure 14:**
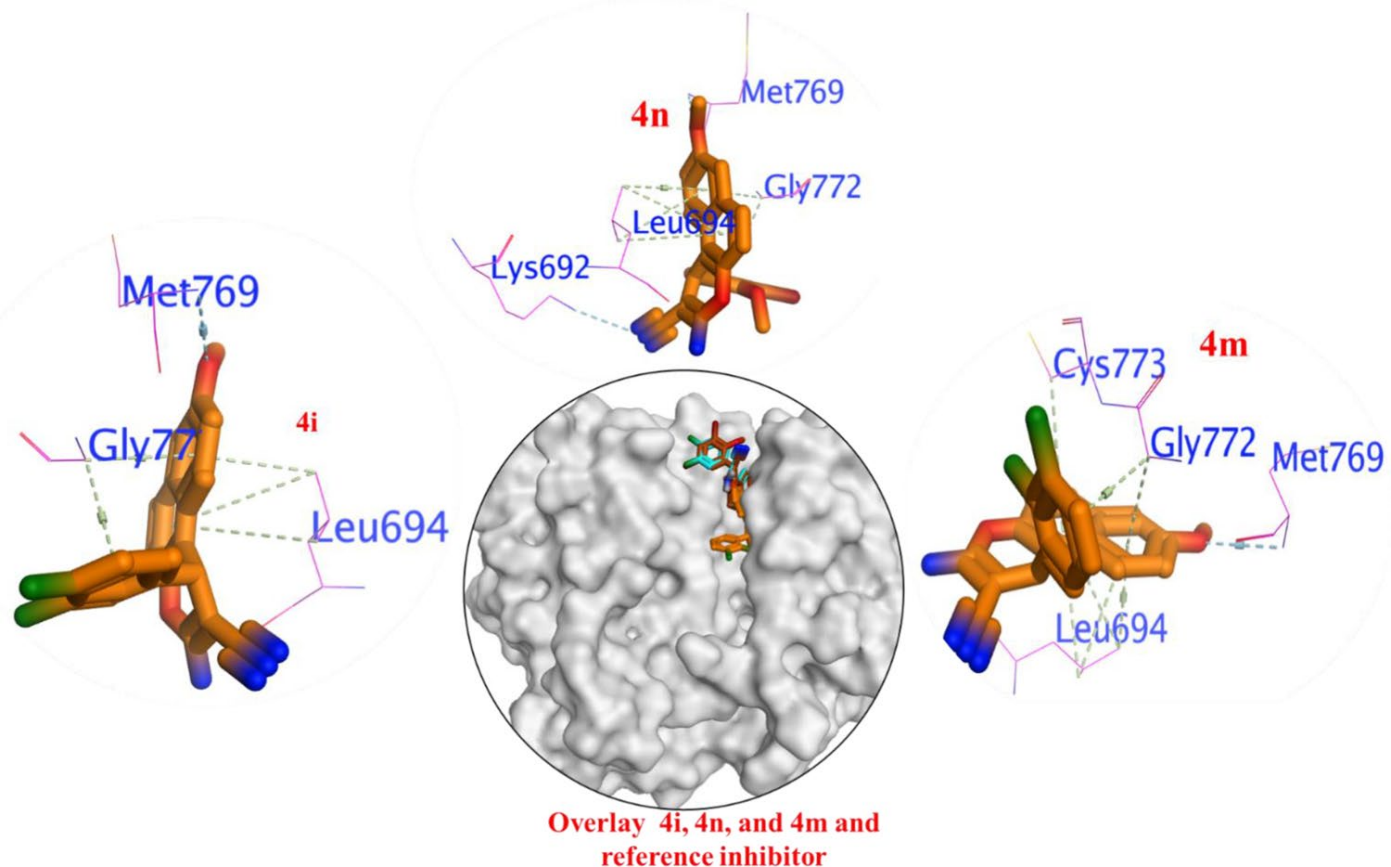
Binding interaction of most active compounds 4i, 4 m, 4n and Erlotinib then superimposed in active site to validate the docking protocol.

The interactions between the eighteen compounds **4a-q** and the active site residues were mainly polar bonds, hydrogen bonding, π − π, and π − H interactions, which contributed to a strong alignment with the enzyme backbone, Fig. 14.

The active molecules **4i, 4 m** and **4n** were attached deeply into the binding pockets, interacting with the vital hydrophobic Met769, GLY772 and LEU694 residues in the same manner of Erlotinib. The most cytotoxic derivatives among the tested compounds were **4i**, **4 m** and **4n**, which showed potent activity against MCF-7, HCT-116 and HepG-2 cancer cell lines. The IC50 values of these derivatives were as follows: **4i**: MCF-7 (0.9 ± 0.01 µg/mL), HCT-116 (1.4 ± 0.15 µg/mL), HepG-2 (11.1 ± 0.17 µg/mL); **4 m**: MCF-7 (0.5 ± 0.21 µg/mL), HCT-116 (0.9 ± 0.97 µg/mL), HepG-2 (0.7 ± 0.06 µg/mL); **4n**: MCF-7 (0.7 ± 0.05 µg/mL), HCT-116 (0.6 ± 0.06 µg/mL), HepG-2 (2.9 ± 0.21 µg/mL). These derivatives also exhibited comparable or higher binding energies than Erlotinib, a known inhibitor of EGFR kinase. The calculated binding energies were: **4i**: − 6.61 kcal/mol; **4 m**: − 6.66 kcal/mol; **4n**: − 6.9 kcal/mol; Erlotinib: − 5.36 kcal/mol.

## Experimental section

### Materials and equipment’s

All chemicals were purchased from Sigma-Aldrich Chemical Co. (Sigma-Aldrich Corp., St. Louis, MO, USA). All melting points were measured with a Stuart Scientific Co. Ltd apparatusare uncorrected. The IR spectra were recorded on a KBr disc on a Jasco FT/IR 460 plus spectrophotometer. The ^1^H NMR (500 MHz) and ^13^C NMR (125 MHz) spectra were measured on BRUKER AV 500 MHz spectrometer in DMSO-d_*6*_ as a solvent, using tetramethylsilane (TMS) as an internal standard, and chemical shifts were expressed as *δ* (ppm). The Microwave apparatus used is Milestone Sr1, Microsynth. Themass spectra were determined on a Shimadzu GC/MS-QP5050A spectrometer. Elemental analysis was carried out at the Regional Centre for Mycology and Biotechnology (RCMP), Al-Azhar University, Cairo, Egypt, and the results were within ± 0.25%. Reaction courses and product mixtures were routinely monitored by thin layer chromatography (TLC) on silica gel precoated F_254_ Merck plates.

### General procedure for synthesis of 1H-benzo[f]chromene derivatives (4a-q)

A reaction mixture of 6-methoxy-2-naphthol (**1**) (0.01 mol), different aromatic aldehydes (**2a-q**) (0.01 mol), malononitrile (**3**) (0.01 mol) and piperidine (0.5 ml) in ethanol (30 ml) was heated under 60 W Ultrasonic irradiation at ambient temperature. After completion of the reaction, the reaction mixture was cooled to room temperature and the precipitated solid was filtered off, washed with methanol, and was recrystallized from ethanol or ethanol/benzene. The physical and spectral data of compounds **4a-q** are as follows:

### 3-Amino-1-(4-fluorophenyl)-8-methoxy-1*H*-benzo[*f*]chromene-2-carbonitrile (4a)

Pale yellow needles from ethanol; yield 91%; m.p. 255–256 ℃ (Literature procedure^25^, Microwave irradiation conditions, yield 87%; m.p. 255–256 ℃), ^1^H NMR *δ*: 7.85–7.07 (m, 9H, Ar), 6.95 (bs, 2H, NH_2_), 5.32 (s, 1H, H-1), 3.83 (s, 3H, OCH_3_); ^13^C NMR *δ*: 159.78 (C-3), 156.45 (C-8), 145.27 (C-4a), 132.22 (C-6a), 128.73 (C-10a), 126.29 (C-6), 125.06 (C-10), 120.43 (C-10b), 119.07 (C-9), 117.11 (C-7), 115.66 (CN), 107.24 (C-5), 57.64 (C-2), 55.19 (CH_3_), 37.30 (C-1), 161.73, 142.01, 128.79, 115.47, 115.30 (Ar); m/z (%): 346 (M^+^, 10.03) with a base peak at 251 (100).

### 3-Amino-1-(2-chlorophenyl)-8-methoxy-1*H*-benzo[*f*]chromene-2-carbonitrile (4b)

Colorless needles from ethanol; yield 89%; m.p. 265–266 °C (Literature procedure^33^, Microwave irradiation conditions, yield 82%; m.p. 265–266 ℃); ^1^H-NMR *δ*: 7.87–6.99 (m, 9H, Ar), 7.00 (bs, 2H, NH_2_), 5.67 (s, 1H, H-1), 3.82 (s, 3H, OCH_3_); ^13^C-NMR *δ*: 159.96 (C-3), 156.49 (C-8), 145.64 (C-4a), 130.97 (C-6a), 129.50 (C-10a), 128.15 (C-6), 125.01 (C-10), 119.87 (C-10b), 119.35 (C-9), 117.09 (C-7), 114.92 (CN), 107.57 (C-5), 56.11 (C-2), 55.21 (CH3), 35.17 (C-1), 142.66, 132.14, 130.00, 128.58, 128.44, 124.09 (Ar); MS m/z (%): 364 (M^+^  + 2, 4.65), 362 (M^+^, 13.54) with a base peak at 251 (100).

### 3-Amino-1-(3-chlorophenyl)-8-methoxy-1*H*-benzo[*f*]chromene-2-carbonitrile (4c)

Colorless needles from ethanol; yield 88%; m.p. 216–217 °C (Literature procedure^33^, Microwave irradiation conditions, yield 86%; m.p. 215–216° ℃),^1^H-NMR *δ*: 7.87–7.11 (m, 9H, Ar), 7.03 (bs, 2H, NH_2_), 5.36 (s, 1H, H-1), 3.83 (s, 3H, OCH_3_); ^13^C-NMR *δ*: 159.94 (C-3), 156.52 (C-8), 148.19 (C-4a), 132.21 (C-6a), 128.48 (C-10a), 126.63 (C-6), 124.99 (C-10), 120.31 (C-10b), 119.21 (C-9), 117.11 (C-7), 115.11 (CN), 107.35 (C-5), 57.18 (C-2), 55.20 (CH3), 37.60 (C-1), 145.36, 133.18, 130.66, 126.61, 125.65 (Ar); MS m/z (%): 364 (M^+^ + 2, 1.24), 362 (M^+^ , 3.58) with a base peak at 208 (100).

### 3-Amino-1-(4-chlorophenyl)-8-methoxy-1*H*-benzo[*f*]chromene-2-carbonitrile (4d)

Pale yellow crystals from ethanol; yield 91%; m.p. 246–247 °C (Literature procedure^25^, Microwave irradiation conditions, yield 89%; m.p. 247–248 ℃); ^1^H NMR *δ*: 7.86–7.10 (m, 9H, Ar), 7.01 (bs, 2H, NH_2_), 5.33 (s, 1H, H-1), 3.83 (s, 3H, OCH_3_); ^13^C NMR *δ*: 159.81 (C-3), 156.47 (C-8), 145.29 (C-4a), 132.21 (C-6a), 128.65 (C-10a), 128.39 (C-6), 125.03 (C-10), 120.37 (C-10b), 119.12 (C-9), 117.11 (C-7), 115.33 (CN), 107.31 (C-5), 57.26 (C-2), 55.20 (CH3), 37.39 (C-1), 144.73, 131.11, 128.77, 124.98 (Ar); MS m/z (%): 364 (M^+^  + 2, 3.39), 362 (M^+^, 11.44) with a base peak at 111 (100).

### 3-Amino-1-(4-bromophenyl)-8-methoxy-1*H*-benzo[*f*]chromene-2-carbonitrile (4e)

Pale yellow crystals from ethanol; yield 92%; m.p. 261–262 °C (Literature procedure^25^, Microwave irradiation conditions, yield 88%; m.p. 261–262 ℃); ^1^H NMR *δ*: 7.86–7.10 (m, 9H, Ar), 7.02 (bs, 2H, NH_2_), 5.32 (s, 1H, H-1), 3.83 (s, 3H, OCH_3_); ^13^C NMR *δ*: 159.80 (C-3), 156.47 (C-8), 145.29 (C-4a), 132.21 (C-6a), 128.40 (C-10a), 128.29 (C-6), 125.02 (C-10), 120.37 (C-10b), 119.13 (C-9), 117.10 (C-7), 115.25 (CN), 107.32 (C-5), 57.20 (C-2), 55.20 (CH_3_), 37.48 (C-1), 145.14, 131.57, 129.16, 119.61 (Ar); MS m/z (%): 408 (M^+^  + 2, 1.30), 406 (M^+^, 1.46) with a base peak at 75 (100).

### 3-Amino-1-(4-iodophenyl)-8-methoxy-1*H*-benzo[*f*]chromene-2-carbonitrile (4f)

Pale yellow crystals from ethanol; yield 89%; m.p. 227–228 °C (Literature procedure^33^, Microwave irradiation conditions, yield 86%; m.p. 227–228 ℃); ^1^ H-NMR *δ*: 7.85–6.99 (m, 9H, ar), 6.98 (bs, 2H, NH_2_), 5.27 (s, 1H, H-1), 3.82 (s, 3H, OCH_3_); ^13^ C-NMR *δ*: 159.80 (C-3), 156.48 (C-8), 145.31 (C-4a), 132.20 (C-6a), 128.37 (C-10a), 125.01 (C-6), 120.37 (C-10), 119.11 (C-10b), 117.08 (C- 7,9), 115.22 (CN), 107.35 (C-5), 57.29 (C-2), 55.21 (CH_3_), 37.69 (C-1), 145.54, 137.43, 129.32, 92.40 (Ar); MS m/z (%): 454 (M^+^, 100).

### 3-Amino-1-(2,4-difluorophenyl)-8-methoxy-1*H*-benzo[*f*]chromene-2-carbonitrile (4g)

Colorless crystals from ethanol; yield 82%; m.p. 300–301 °C (Literature procedure^33^, Microwave irradiation conditions, yield 81%; m.p. 301–302° ℃); ^1^H-NMR *δ*: 7.83–7.05 (m, 8H, Ar), 7.09 (bs, 2H, NH_2_), 5.67 (s, 1H, H-1), 3.82 (s, 3H, OCH_3_); ^13^C-NMR *δ*: 160.71 (C-3), 149.61 (C-8), 145.61 (C-4a), 131.44 (C-6a), 129.11 (C-10a), 128.39 (C-6), 123.52 (C-10), 120.18 (C-9), 120.04 (C-7), 119.92 (C-10b), 116.94 (CN), 107.57 (C-5), 55.19 (C-2), 53.58 (CH_3_), 28.39 (C-1), 161.40, 156.14, 129.39, 125.59, 116.64, 112.82 (Ar); MS m/z (%): 364 (M^+^, 61.46) with a base peak at 251 (100).

### 3-Amino-1-(2,6-difluorophenyl)-8-methoxy-1*H*-benzo[*f*]chromene-2-carbonitrile (4h)

Yellow crystals from ethanol; yield 88%; m.p. 295–296 °C (Literature procedure^51^, Microwave irradiation conditions, yield 86%; m.p. 295–296 ℃), ^1^H NMR *δ*: 7.03–7.83 (m, 7H, Ar), 7.09 (bs, 2H, NH_2_, exchangeable with D_2_O), 5.61 (s, 1H, CH), 3.82 (s, 3H, CH_3_). ^13^C NMR *δ*: 160.61 (C-3),145.71 (C-8), 131.93 (C-4a), 129.59 (C-6a), 129.52 (C-10a), 128.44 (C-6), 123.11 (C-10), 120.18 (C-9), 120.04 (C-7), 119.92 (C-10b), 116.94 (CN), 107.57 (C-5), 55.19 (C-2), 53.58 (CH_3_), 28.17 (C-1), 156.40, 161.14, 125.03, 112.82 (Ar), MS m/z (%): 364 (M^+^, 60.23) with a base peak at 251 (100).

### 3-Amino-1-(2,3-dichlorophenyl)-8-methoxy-1*H*-benzo[*f*]chromene-2-carbonitrile (4i)

Colorless needles from ethanol; yield 88%; m.p. 256–257 °C (Literature procedure^33^, Microwave irradiation conditions, yield 87%; m.p. 255–256 ℃), ^1^H-NMR *δ*: 7.88–6.96 (m, 8H, Ar), 7.07 (bs, 2H, NH_2_), 5.74 (s, 1H, H-1), 3.83 (s, 3H, OCH_3_); ^13^ C-NMR *δ*: 160.08 (C-3), 156.53 (C-8), 145.23 (C-4a), 131.98 (C-6a), 129.16 (C-10a), 128.79 (C-6), 124.92 (C-10), 119.77 (C-10b), 119.50 (C-9), 117.09 (C-7), 114.44 (CN), 107.65 (C-5), 55.59 (C-2), 55.21 (CH_3_), 36.22 (C-1), 145.63, 132.16, 129.01, 128.93, 128.64, 123.95 (Ar); MS m/z (%): 400 (M^+^  + 4, 10.10), 398 (M^+^  + 2, 3.37), 396 (M^+^, 12.08) with a base peak at 110 (100).

### 3-Amino-1-(2,4-dichlorophenyl)-8-methoxy-1*H*-benzo[*f*]chromene-2-carbonitrile (4j)

Colorless crystals from ethanol; yield 87%; m.p. 285–286 °C (Literature procedure^52^, Microwave irradiation conditions, yield 83%; m.p. 284–285 ℃), ^1^H NMR *δ*: 7.03–7.83 (*m*, 7H, Ar), 7.09 (bs, 2H, NH_2_, exchangeable with D_2_O), 5.61 (*s*, 1H, CH), 3.82 (*s*, 3H, CH_3_). ^13^C NMR *δ*: ^13^ C-NMR *δ*: 160.61 (C-3), 156.40 (C-8), 145.71 (C-4a), 131.93 (C-6a), 129.59 (C-10a), 129.11 (C-6), 127.98 (C-10),127.03 (C-10b), 126.11 (C-9), 123.04 (C-7), 120.18 (CN), 116.94 (C-5), 55.19 (C-2), 53.58 (CH_3_), 28 17 (C-1), 161.14, 135.75, 134.56, 132.45, 131.05, 128.44 (Ar); MS m/z (%): 400 (M^+^  + 4, 1.31), 398 (M^+^  + 2, 8.02), 396 (M^+^, 13.38) with a base peak at 208 (100).

### 3-Amino-1-(2,5-dichlorophenyl)-8-methoxy-1*H*-benzo[*f*]chromene-2-carbonitrile (4k)

Colorless crystals from ethanol; yield 87%; m.p. 270–271 °C (Literature procedure^42^, Microwave irradiation conditions, yield 82%; m.p. 270–271 ℃); ^1^H NMR *δ*: 7.88–6.96 (m, 8H, Ar), 7.07 (bs, 2H, NH_2_), 5.74 (s, 1H, H-1), 3.83 (s, 3H, OCH_3_); ^13^ C NMR *δ*: 160.08 (C-3), 156.53 (C-8), 145.23 (C-4a), 131.98 (C-6a), 128.79 (C-10a), 128.64 (C-6), 123.95 (C-10), 119.77 (C-10b), 119.50 (C-9), 117.09 (C-7), 114.44 (CN), 107.65 (C-5), 55.59 (C-2), 55.21 (CH_3_), 36.23 (C-1), 145.63, 132.16, 129.15, 129.01, 128.93, 124.92 (Ar); MS m/z (%): 400 (M^+^ + 4, 45.79), 398 (M^+^ + 2, 15.27), 396 (M^+^ , 53.43) with a base peak at 180 (100).

### Amino-1-(2,6-dichlorophenyl)-8-methoxy-1*H*-benzo[*f*]chromene-2-carbonitrile (4l)

Colorless needles from ethanol; yield 88%; m.p. 313–314 °C (Literature procedure^33^, Microwave irradiation conditions, yield 85%; m.p. 314–315 ℃); ^1^H-NMR *δ*: 7.84–7.09 (m, 8H, Ar), 7.05 (bs, 2H, NH_2_), 6.09 (s, 1H, H-1), 3.82 (s, 3H, OCH_3_); ^13^C-NMR *δ*: 160.41 (C-3), 156.22 (C-8), 146.39 (C-4a), 134.33 (C-6a), 132.01 (C-10a), 128.86 (C-6), 125.22 (C-10), 23.83 (C-10b), 119.56 (C-9), 119.18 (C-7), 116.78 (CN), 107.69 (C-5), 55.18 (C-2), 52.59 (CH_3_), 5.19 (C-1), 137.45, 135.04, 130.96, 129.57 (Ar); MS m/z (%): 400 (M^+^ + 4, 6.19), 398 (M^+^ + 2, 2.09), 396 (M^+^, 7.88) with a base peak at 251 (100).

### 3-Amino-1-(3,4-dichlorophenyl)-8-methoxy-1*H*-benzo[*f*]chromene-2-carbonitrile (4m)

Colorless crystals from ethanol; yield 87%; m.p. 241–242 °C (Literature procedure^33^, Microwave irradiation conditions, yield 85%; m.p. 240–241 ℃); ^1^H-NMR *δ*: 7.88–6.96 (m, 8H, Ar), 7.08 (bs, 2H, NH_2_), 5.76 (s, 1H, H-1), 3.84 (s, 3H, OCH_3_); ^13^C-NMR *δ*: 160.08 (C-3), 156.53 (C-8), 145.63 (C-4a), 129.01 (C-6a), 128.79 (C-10a), 128.64 (C-6), 123.95 (C-10), 119.77 (C-10b), 119.50 (C-9), 117.09 (C-5), 114.44 (CN), 107.65 (C-7), 55.59 (C-2), 55.21 (CH_3_), 36.23 (C-1), 145.23, 132.16, 131.98, 129.15, 128.93, 128.79, 124.92 (Ar); MS m/z (%): 400 (M^+^ + 4, 1.49), 398 (M^+^ + 2, 0.47), 396 (M + , 1.79) with a base peak at 208 (100).

### 3-amino-1-(3,5-dibromo-2-methoxyphenyl)-8-methoxy-1*H*-benzo[*f*]chromene-2-carbonitrile (4n)

Colorless crystals from ethanol; yield 87%; m.p. 241–242 °C (Literature procedure^53^, Microwave irradiation conditions, yield 85%; m.p. 240–241 ℃); ^1^H-NMR *δ*: 7.18–7.85 (*m*, 7H, Ar), 7.12 (bs, 2H, NH_2_, exchangeable with D_2_O), 5.50 (*s*, 1H, CH), 3.83 (*s*, 3H, CH_3_), 3.75 (*s*, 3H, CH_3_). ^13^ C NMR *δ*: 160.54 (C-3), 156.56 (C-8), 145.21 (C-4a), 134.24 (C-6a), 132.07 (C-10a), 131.63 (C-6), 125.07 (C-10), 120.62 (C-10b), 119.38 (C-9), 118.16 (C-5), 118.33 (CN), 107.55 (C-7), 61.78 (C-2), 56.04 (CH_3_), 55.21 (CH_3_), 33.84 (C-1), 153.10, 142.68, 128.55, 124.25, 117.25, 116.96 (Ar); MS m/z (%): 518 (M^+^ + 4, 18.49), 516 (M^+^ + 2, 38.49), 514 (M + , 20.73) with a base peak at 252 (100).

### 3-amino-1-(2,3,4-trimethoxyphenyl)-8-methoxy-1*H*-benzo[*f*]chromene-2-carbonitrile (4o)

Colorless crystals from ethanol; yield 87%; m.p. 240–241 °C (Literature procedure^33^, Microwave irradiation conditions, yield 84%; m.p. 239–240 ℃); ^1^H NMR *δ*: 7.85–6.95 (m, 7H, Ar), 6.96 (bs, 2H, NH_2_), 5.26 (s, 1H, H-1), 3.79 (s, 3H, OCH_3_), 3.66 (s, 6H, 2OCH_3_), 3.60 (s, 3H, OCH_3_); ^13^C NMR *δ*: 159.72 (C-3), 158.00 (C-9), 147.20 (C-4a), 131.83 (C-10a), 129.94 (C-7), 129.05 (C-6), 125.94 (C-6a), 120.53 (C-10b), 116.99 (C-5), 114.88 (CN), 114.02 (C-8), 103.21 (C-10), 59.91 (CH_3_), 57.88 (C-2), 56.13 (CH_3_), 55.80 (CH_3_), 55.11 (CH_3_), 38.29 (C-1), 152.90, 141.62, 136.18, 112.67, 104.65 (Ar); MS m/z (%): 418 (M^+^, 77.17) with a base peak at 387 (100).

### 3-amino-1-(3,4,5-trimethoxyphenyl)-8-methoxy-1*H*-benzo[*f*]chromene-2-carbonitrile (4p)

Colorless crystals from ethanol; yield 89%; m.p. 258–259 °C (Literature procedure^33^, Microwave irradiation conditions, yield 84%; m.p. 259–260 ℃); ^1^H NMR *δ*: 7.78–6.60 (m, 7H, Ar), 6.92 (bs, 2H, NH_2_), 5.35 (s, 1H, H-1), 3.81 (s, 3H, OCH_3_), 3.79 (s, 3H, OCH_3_), 3.72 (s, 3H, OCH_3_), 3.66 (s, 3H, OCH_3_); ^13^C NMR *δ*: 160.60 (C-3), 156.80 (C-8), 145.70 (C-4a), 131.85 (C-10a), 128.23 (C-6), 125.75 (C-6a), 125.00 (C-10), 121.53 (C-10b), 119.46 (C-5), 117.55 (C-9), 116.86 (CN), 107.60 (C-10), 61.75 (CH_3_), 60.62 (CH_3_), 57.62 (C-2), 56.05 (CH_3_), 55.57 (CH_3_), 33.04 (C-1), 152.49, 150.24, 141.71, 132.45, 123.80, 108.77 (Ar); ^13^ C NMR-DEPT spectrum at 135° CH, CH_3_ [positive (up)], CH_2_ [negative (down)], revealed the following signals at *δ*: 128.23 (C-6 ↑), 125.00 (C-10 ↑), 123.80 (Ar ↑), 119.46 (C-5), 117.55 (C-9), 108.77 (Ar ↑), 107.60 (C-10 ↑), 61.75 (CH_3_ ↑), 60.62 (CH_3_ ↑), 56.05 (CH_3_ ↑), 55.57 (CH_3_ ↑), 33.04 (C-1 ↑); MS m/z (%): 418 (M^+^, 13.93) with a base peak at 40 (100).

### 3-amino-1-(2,3,5-trichlorophenyl)-8-methoxy-1*H*-benzo[*f*]chromene-2-carbonitrile (4q)

Colorless crystals from ethanol; yield 88%; m.p. 259–260 °C (Literature procedure^33^, Microwave irradiation conditions, yield 84%; m.p. 259–260 ℃); MS m/z (%): 436 (M^+^  + 6, 3.91), 434 (M^+^  + 4, 33.80), 432 (M^+^  + 2, 96.27), 430 (M^+^, 100).

### General procedure for synthesis of 2-(4-halobenzylidene)malononitriles (7a,d,e)

The interaction of 6-cyanonaphthalen-2-ol (**5**) with different aromatic aldehydes (**2a,d,e**) (0.01 mol), malononitrile (**3**) in absolute ethanol/piperidine solution utilizing stirring at room temperature for 2 h, reflux for 2 h, Microwave irradiation conditions for 2 min. 400 W at 140 °C or 60 W Ultrasonic irradiation was unsuccessful, 3-amino-1-aryl-1*H*-benzo[*f*]chromene-2,8-dicarbonitrile (**6a,d,e**) not formed. After completion of the reaction, the reaction mixture was cooled to room temperature and the precipitated solid was filtered off, washed with methanol, and was recrystallized from ethanol to afforded the 2-(4-halobenzylidene)malononitriles (**7a,d,e**) (m.p., mixed m.p. and identical IR). The physical and spectral data of compounds **7a,d,e** are as follows:

### 2-(4-Flouroenzylidene)malononitrile (7a)

Yellow crystals from ethanol; yield 96%; m.p. 126–127 °C (Literature procedure^61^, yield 97%; m.p. 125–126 ℃).

### 2-(4-Chlorobenzylidene)malononitrile (7d)

Colourless needles from ethanol; yield 95%; m.p. 162–163 °C (Literature procedure^61^, yield 98%; m.p. 90–91 ℃).

### 2-(4-Bromobenzylidene)malononitrile (7e)

Colourless needles from ethanol; yield 97%; m.p. 164–165 °C (Literature procedure^61^, yield 99%; m.p. 165–166 ℃).

### Biology

#### Antibadterial screening

Compound **4a-q** was screened for its in vitro antimicrobial activities against pathogenic bacteria species using the standard antibiotics Ampicillin, Gentamycin and Ketoconazole as reference drugs as reported previously^54^. The minimum inhibitory concentration (MIC) was determined as previously reported^55^. The antimicrobial activities were performed at the Regional Centre for Mycology & Biotechnology (RCMP), Al-Azhar University.

#### Cell culture and cytotoxicity evaluation using viability assay

Compounds **4a-q** was initially evaluated for in vitro antitumor activity against three different human cell lines: MCF-7, HCT-116 and HepG-2. In-vitro cytotoxicity evaluation was performed at the Regional Center for Mycology & Biotechnology (RCMP), Al-Azhar University under different concentrations (50, 25, 12.5, 6.25, 3.125, 1.56 and 0 µg/mL); vinblastine and doxorubicin are used as reference cytotoxic compounds. The measurements of cell growth and the *in-vitro* cytotoxicity evaluation were determined using viability assay as described in literature^56^ and the result was cited in Table 3 and Fig. 8.

## Conclusions

In summary, a library of halogenated 3-amino-1-aryl-8-methoxy-1*H*-benzo[*f*]chromene-2-carbonitriles (**4a-q**) was synthesized and evaluated for their anticancer potential. The majority of these compounds demonstrated significant antiproliferative activities against the tested cancer cell lines, MCF-7, HCT-116 and HepG-2. The pyran derivatives **4 m,n,j** with a double chlorine at 3,4-positions, a double bromine at 3,5-positions with a single methoxy group at 2-position and a double chlorine at 2,4-positions of phenyl ring and, to lesser extent, other pyran derivatives with dihalogenated phenyl ring (**4i** and **4 g**) exhibited the highest cytotoxicity against three human cancer cell lines MCF-7, HCT-116 and HepG-2, while compound **4 g** exhibited the highest antimicrobial activity than the other pyran derivatives (**4a-f,g-q**). In addition, MIC was assessed and screened for **4 g**, revealing bactericidal effects. On the other hand, the SAR study has been performed on the desired compounds and elucidated that the position and the type of the substituent on the phenyl group at the 1-position of the 1*H*-benzo[*f*]chromene moiety enhanced their antitumor activities. In addition the presence of the lipophilic electron-withdrawing group was essential for the activity.

### Supplementary Information

Supplementary Figures.

## Data Availability

The authors declare that the data supporting the finding of this study are available in supplementary files.
