# Supplementary material for: Targeted potent antimicrobial and antitumor oxygen-heterocyclic-based pyran analogues: synthesis and computational studies
Source: Sci Rep. 2024 Apr 29;14:9862. doi: 10.1038/s41598-024-59193-2 (PMC11058275; doi:10.1038/s41598-024-59193-2)
Supplement: Supplementary file 1 — Supplementary Figures. [file 41598_2024_59193_MOESM1_ESM.docx]

**Targeted potent antimicrobial and antitumor oxygen-heterocyclic-based pyran analogues: Synthesis and computational studies**

**Ashraf H.F. Abd El-Wahab ^a^, Rita M. Borik ^a^, Al-Anood M. Al-Dies,^b^ Ahmed M. Fouda ^c^,**  **Hany M. Mohamed ^a^, Raafat A. El-Eisawy ^d,e^, Mohamed H. Sharaf ^f^ , Abdullah Y. A. Alzahrani ^g^, Ahmed A. Elhenawy ^d,h^, Ahmed M. El-Agrody ^d,^***

*^a^ Department of Chemistry, college of Science, Jazan University, B.O. Box 114, Jazan 45142, Kingdom of Saudi Arabia*

*^b^Chemistry Department, Umm Al-Qura University,Al-Qunfudah University College, Al-Qunfudah 21912, Saudi Arabia*

*^c^ Chemistry Department, Faculty of Science, King Khalid University, Abha 61413, Saudi Arabia*

***^d^*** *Chemistry Department, Faculty of Science, Al-Azhar University, Nasr City 11884, Cairo, Egypt*

*^e^ Department of Chemistry, Faculty of Science, Al-Baha University, Al-Baha, 65528, Saudi Arabia*

*^f^ Department of Botany and Microbiology, Faculty of Science, Al-Azhar University, Cairo 11884, Egypt*

*^g^ Department of Chemistry, Faculty of Science and Arts, King Khalid University,Mohail Assir, Saudi Arabia*

*^h^ Chemistry Department, Faculty of Science and Art, AlBaha University, Al Bahah 65731, Saudi Arabia*

*** Corresponding authors: Ahmed M. El-Agrody, elagrody_am@azhar.edu.eg**

| **Table of contents** | **Page** |
| --- | --- |
| **Fig. S1.** ^1^H-NMR spectrum (DMSO-*d_6_*, 500 MHz) of compound **4a**. | S4 |
| **Fig. S2.** Enlarged ^1^H-NMR spectrum (DMSO-*d_6_*, 500 MHz) of compound **4a**. | S5 |
| **Fig. S3.** ^13^C-NMR spectrum (DMSO-*d_6_*, 125 MHz) of compound **4a**. | S6 |
| **Fig. S4.** ^1^H-NMR spectrum (DMSO-*d_6_*, 500 MHz) of compound **4b**. | S7 |
| **Fig. S5.** Enlarged ^1^H-NMR spectrum (DMSO-*d_6_*, 500 MHz) of compound **4b**. | S8 |
| **Fig. S6.** ^13^C-NMR spectrum (DMSO-*d_6_*, 125 MHz) of compound **4b**. | S9 |
| **Fig. S7.** ^1^H-NMR spectrum (DMSO-*d_6_*, 500 MHz) of compound **4c**. | S10 |
| **Fig. S8.** Enlarged ^1^H-NMR spectrum (DMSO-*d_6_*, 500 MHz) of compound **4c**. | S11 |
| **Fig. S9.** ^13^C-NMR spectrum (DMSO-*d_6_*, 125 MHz) of compound **4c**. | S12 |
| **Fig. S10.** ^1^H-NMR spectrum (DMSO-*d_6_*, 500 MHz) of compound **4d**. | S13 |
| **Fig. S11.** Enlarged ^1^H-NMR spectrum (DMSO-*d_6_*, 500 MHz) of compound **4d**. | S14 |
| **Fig. S12.** ^13^C-NMR spectrum (DMSO-*d_6_*, 125 MHz) of compound **4d**. | S15 |
| **Fig. S13.** ^1^H-NMR spectrum (DMSO-*d_6_*, 500 MHz) of compound **4e**. | S16 |
| **Fig. S14.** Enlarged ^1^H-NMR spectrum (DMSO-*d_6_*, 500 MHz) of compound **4e**. | S17 |
| **Fig. S15.** ^13^C-NMR spectrum (DMSO-*d_6_*, 125 MHz) of compound **4e**. | S18 |
| **Fig. S16.** ^1^H-NMR spectrum (DMSO-*d_6_*, 500 MHz) of compound **4f**. | S19 |
| **Fig. S17.** Enlarged ^1^H-NMR spectrum (DMSO-*d_6_*, 500 MHz) of compound **4f**. | S20 |
| **Fig. S18.** ^13^C-NMR spectrum (DMSO-*d_6_*, 125 MHz) of compound **4f**. | S21 |
| **Fig. S19.** ^1^H-NMR spectrum (DMSO-*d_6_*, 500 MHz) of compound **4g**. | S22 |
| **Fig. S20.** Enlarged ^1^H-NMR spectrum (DMSO-*d_6_*, 500 MHz) of compound **4g**. | S23 |
| **Fig. S21.** ^13^C-NMR spectrum (DMSO-*d_6_*, 125 MHz) of compound **4g**. | S24 |
| **Fig. S22.** ^1^H-NMR spectrum (DMSO-*d_6_*, 500 MHz) of compound **4h**. | S25 |
| **Fig. S23.** ^1^H-NMR spectrum (DMSO-*d_6_*, 500 MHz) of compound **4h**. | S26 |
| **Fig. S24.** ^13^C-NMR spectrum (DMSO-*d_6_*, 500 MHz) of compound **4h**. | S27 |
| **Fig. S25.** ^1^H-NMR spectrum (DMSO-*d_6_*, 500 MHz) of compound **4i**. | S28 |
| **Fig. S26.** Enlarged ^1^H-NMR spectrum (DMSO-*d_6_*, 500 MHz) of compound **4i**. | S29 |
| **Fig. S27.** ^13^C-NMR spectrum (DMSO-*d_6_*, 125 MHz) of compound **4i**. | S30 |
| **Fig. S28.** ^1^H-NMR spectrum (DMSO-*d_6_*, 500 MHz) of compound **4k**. | S31 |
| **Fig. S29.** Enlarged ^1^H-NMR spectrum (DMSO-*d_6_*, 500 MHz) of compound **4k**. | S32 |
| **Fig. S30.** ^13^C-NMR spectrum (DMSO-*d_6_*, 125 MHz) of compound **4k**. | S33 |
| **Fig. S31**. ^1^H-NMR spectrum (DMSO-*d_6_*, 500 MHz) of compound **4l**. | S34 |
| **Fig. S32.** Enlarged ^1^H-NMR spectrum (DMSO-*d_6_*, 500 MHz) of compound **4l**. | S35 |
| **Fig. S33.** ^13^C-NMR spectrum (DMSO-*d_6_*, 125 MHz) of compound **4l**. | S36 |
| **Fig. S34.** ^1^H-NMR spectrum (DMSO-*d_6_*, 500 MHz) of compound **4m**. | S37 |
| **Fig. S35.** Enlarged ^1^H-NMR spectrum (DMSO-*d_6_*, 500 MHz) of compound **4m**. | S38 |
| **Fig. S36.** ^13^C-NMR spectrum (DMSO-*d_6_*, 125 MHz) of compound **4m**. | S39 |
| **Fig. S37.** ^1^H-NMR spectrum (DMSO-*d_6_*, 500 MHz) of compound **4n**. | S40 |
| **Fig. S38.** Enlarged ^1^H-NMR spectrum (DMSO-*d_6_*, 500 MHz) of compound **4n**. | S41 |
| **Fig. S39.** ^13^C-NMR spectrum (DMSO-*d_6_*, 125 MHz) of compound **4n**. | S42 |
| **Fig. S40.** ^1^H-NMR spectrum (DMSO-*d_6_*, 500 MHz) of compound **4p**. | S43 |
| **Fig. S41.** ^13^C-NMR spectrum (DMSO-*d_6_*, 125 MHz) of compound **4p**. | S44 |
| **Fig. S42.** DEPT spectrum (DMSO-*d_6_*, 125 MHz) of compound **4p**. | S45 |
| **Fig. S43.** ^1^H-NMR spectrum (DMSO-*d_6_*, 500 MHz) of compound **4q**. | S46 |
| **Fig. S44.** ^13^C-NMR spectrum (DMSO-*d_6_*, 125 MHz) of compound **4q**. | S47 |
| **Fig. S45.** APT spectrum (DMSO-*d_6_*, 125 MHz) of compound **4q**. | S48 |


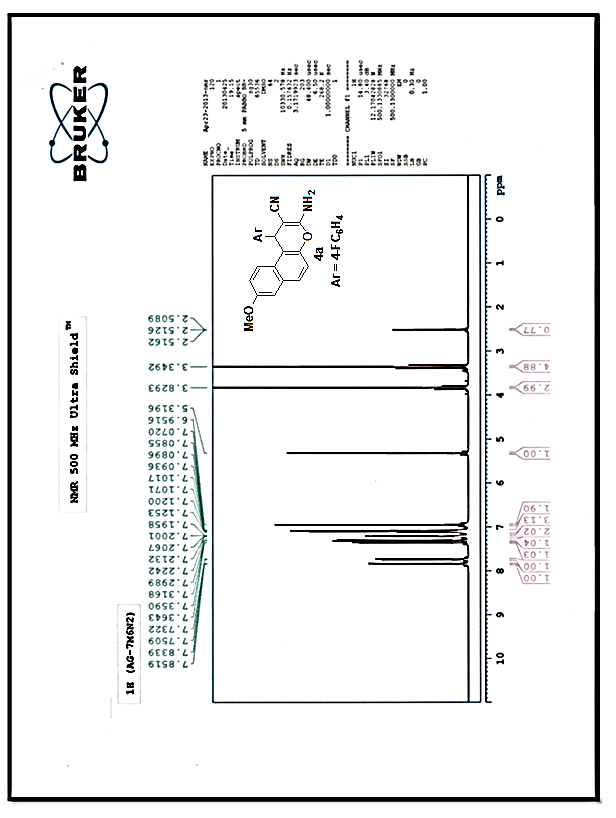


**Fig. S1**. ^1^H-NMR spectrum (DMSO-*d_6_*, 500 MHz) of compound **4a.**


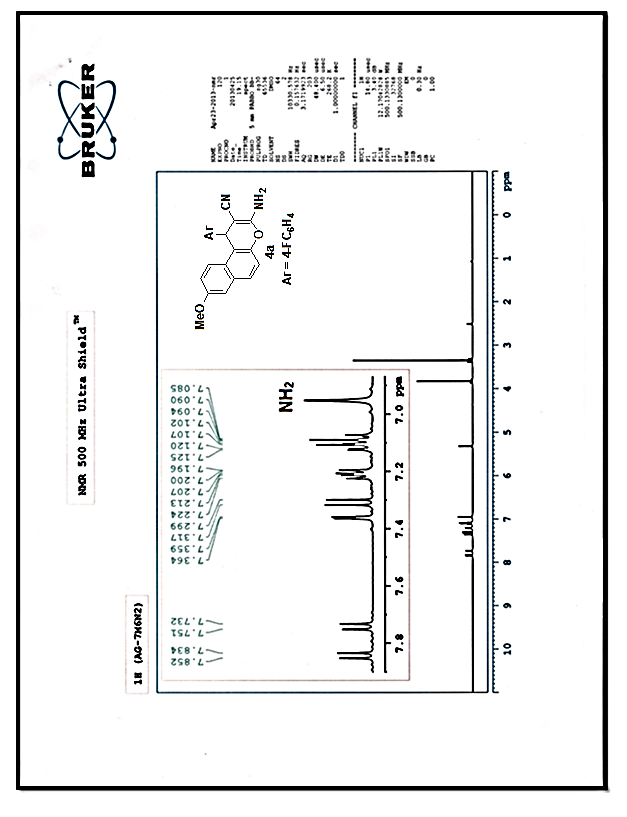


**Fig. S2.** Enlarged ^1^H-NMR spectrum (DMSO-*d_6_*, 500 MHz) of compound **4a.**


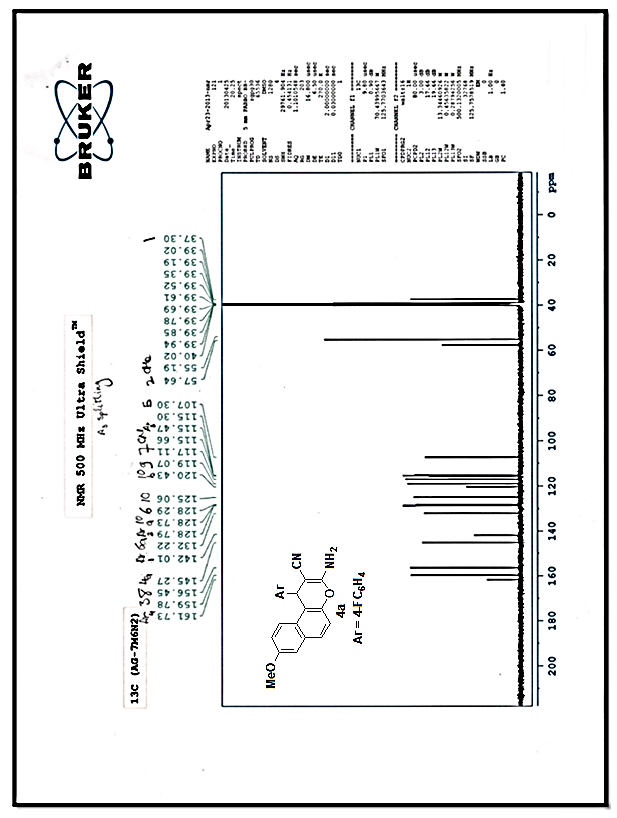


**Fig. S3.** ^13^C-NMR spectrum (DMSO-*d_6_*, 125 MHz) of compound **4a.**


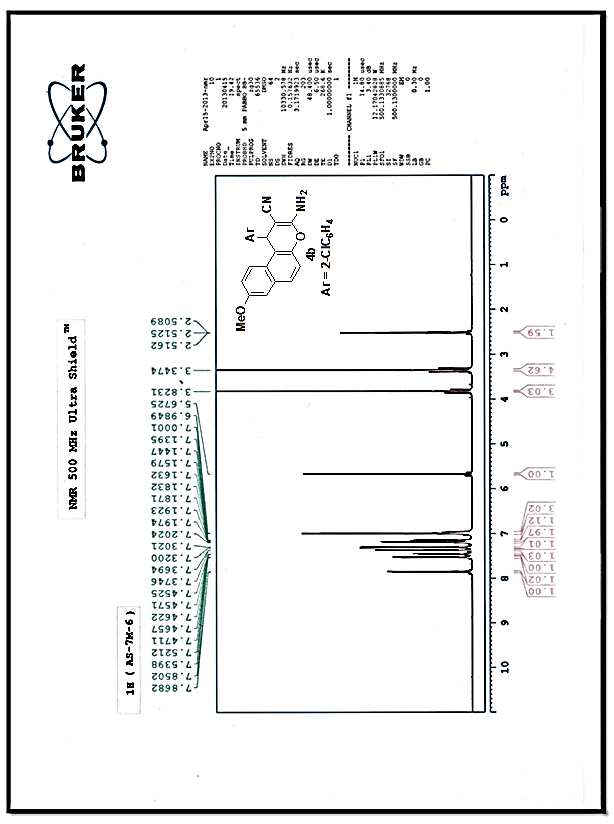


**Fig. S4.** ^1^H-NMR spectrum (DMSO-*d_6_*, 500 MHz) of compound **4b.**


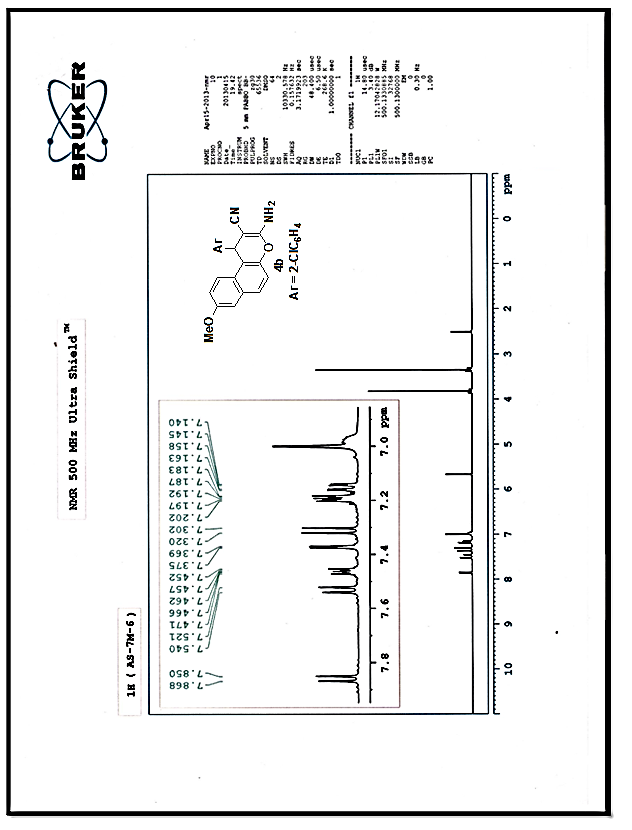


**Fig. S5.** Enlarged ^1^H-NMR spectrum (DMSO-*d_6_*, 500 MHz) of compound **4b.**


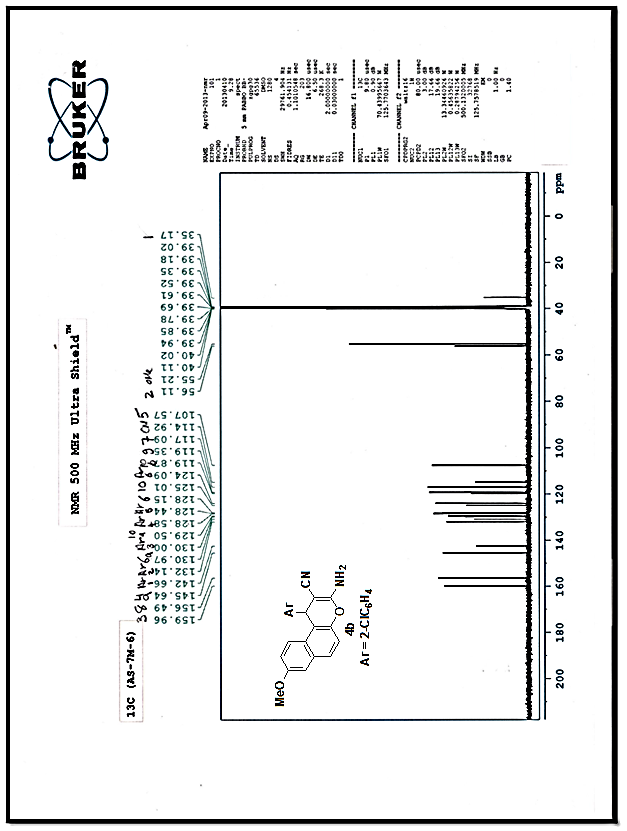


**Fig. S6.** ^13^C-NMR spectrum (DMSO-*d_6_*, 125 MHz) of compound **4b.**


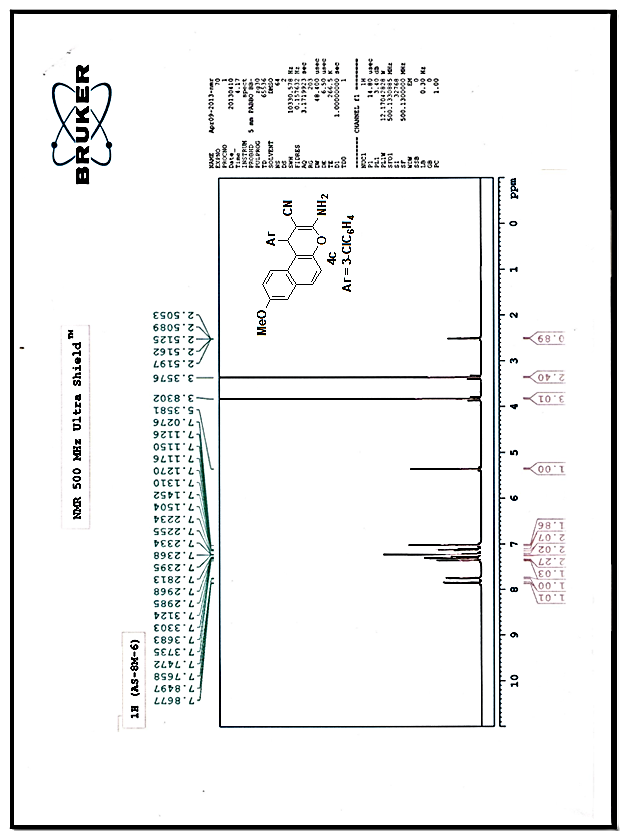


**Fig. S7.** ^1^H-NMR spectrum (DMSO-*d_6_*, 500 MHz) of compound **4c.**


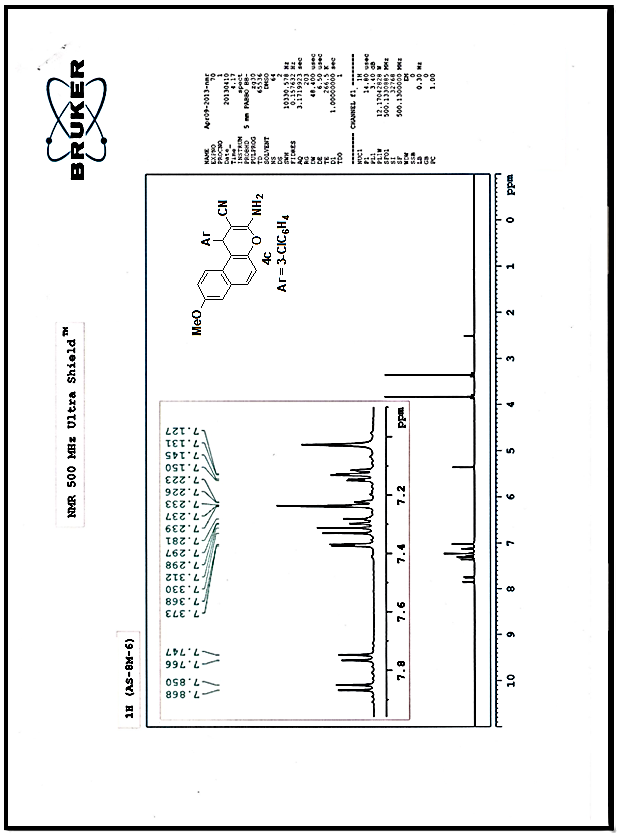


**Fig. S8.** Enlarged ^1^H-NMR spectrum (DMSO-*d_6_*, 500 MHz) of compound **4c.**


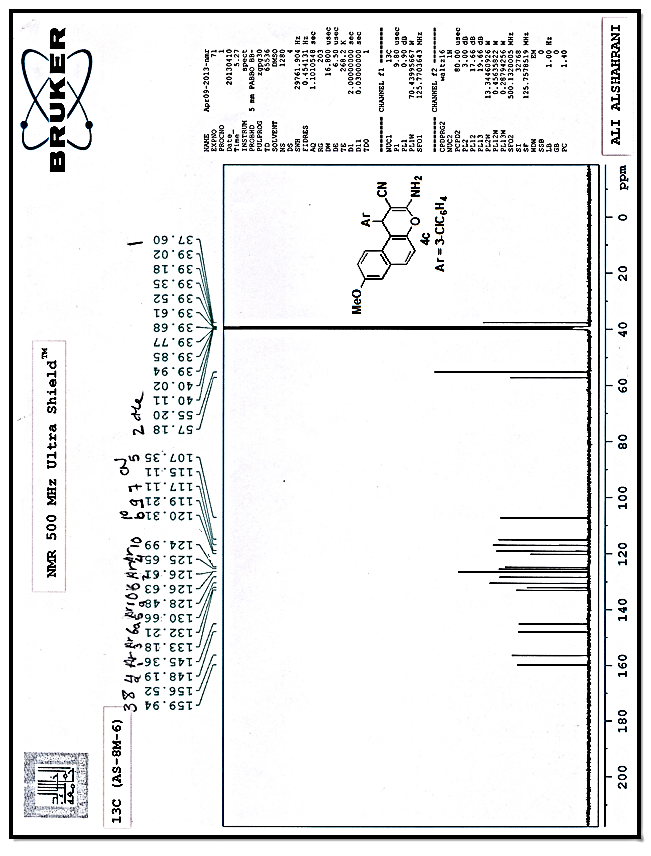


**Fig. S9.** ^13^C-NMR spectrum (DMSO-*d_6_*, 125 MHz) of compound **4c.**


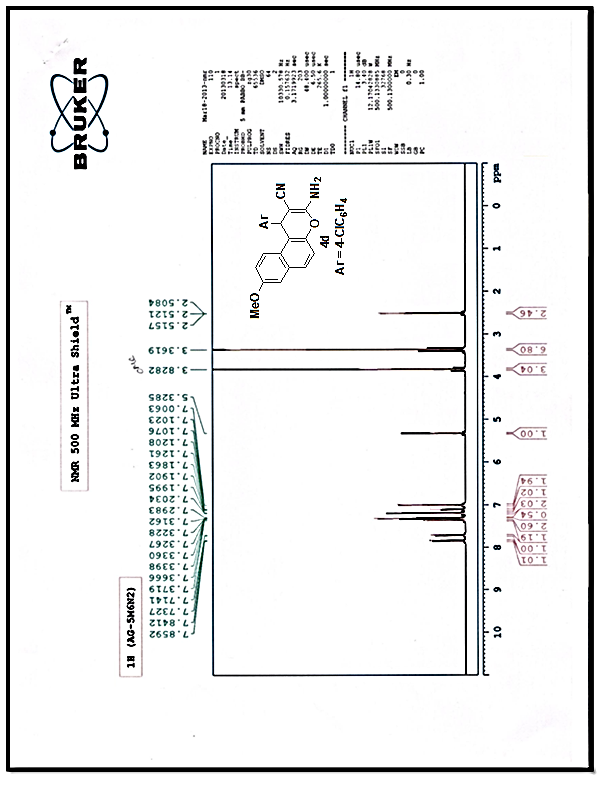


**Fig. S10.** ^1^H-NMR spectrum (DMSO-*d_6_*, 500 MHz) of compound **4d.**


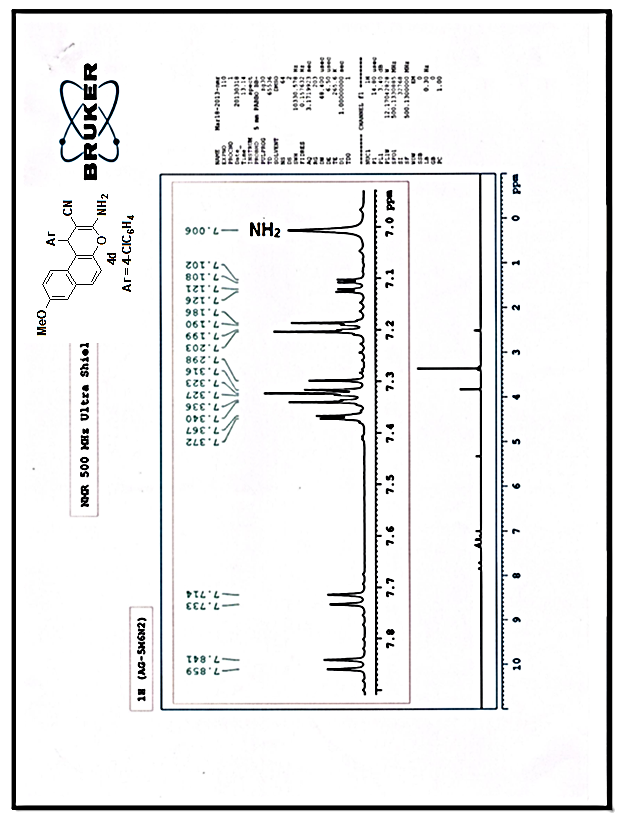


**Fig. S11.** Enlarged ^1^H-NMR spectrum (DMSO-*d_6_*, 500 MHz) of compound **4d.**


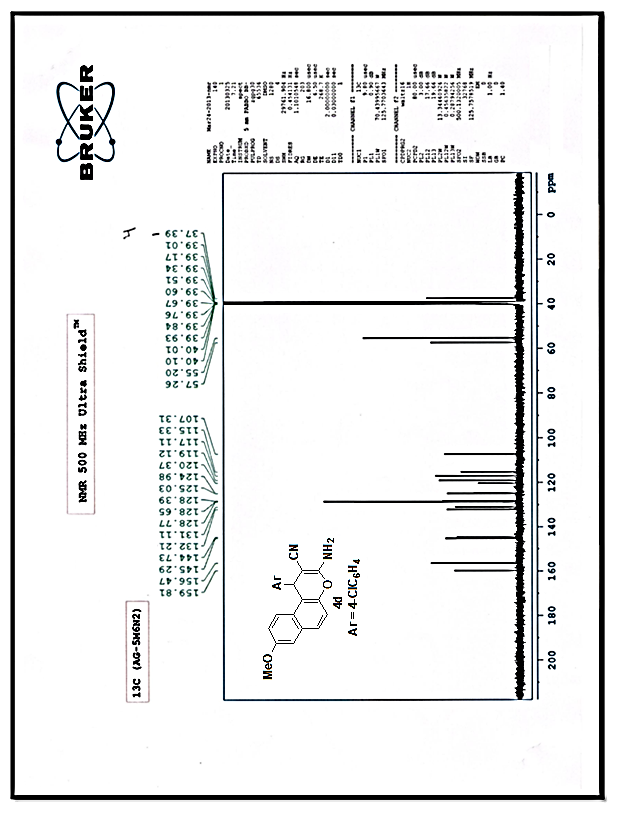


**Fig. S12.** ^13^C-NMR spectrum (DMSO-*d_6_*, 125 MHz) of compound **4d.**


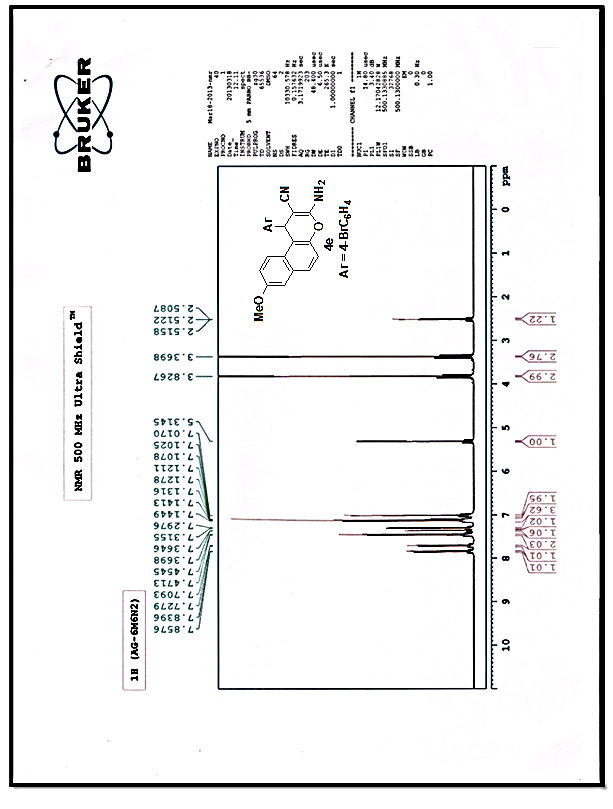


**Fig. S13.** ^1^H-NMR spectrum (DMSO-*d_6_*, 500 MHz) of compound **4e.**


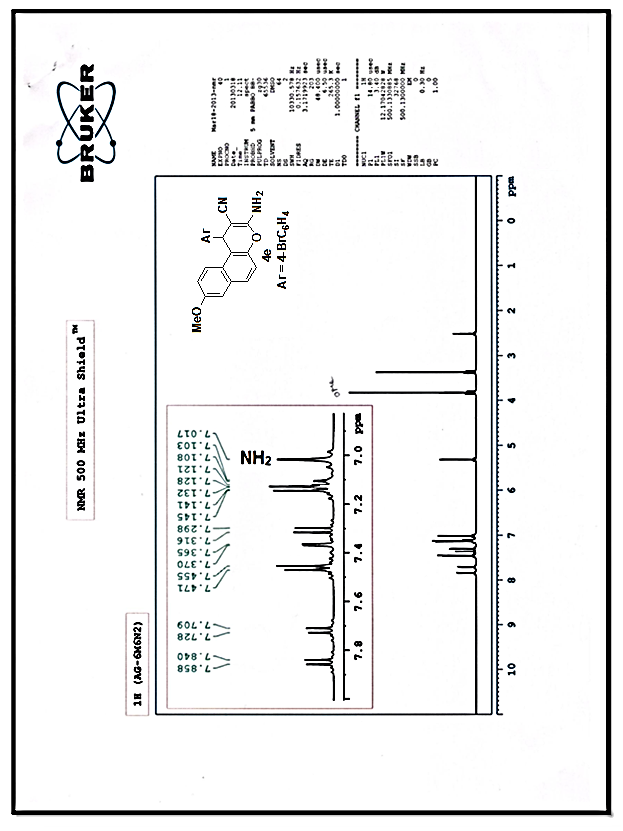


**Fig. S14.** Enlarged ^1^H-NMR spectrum (DMSO-*d_6_*, 500 MHz) of compound **4e.**


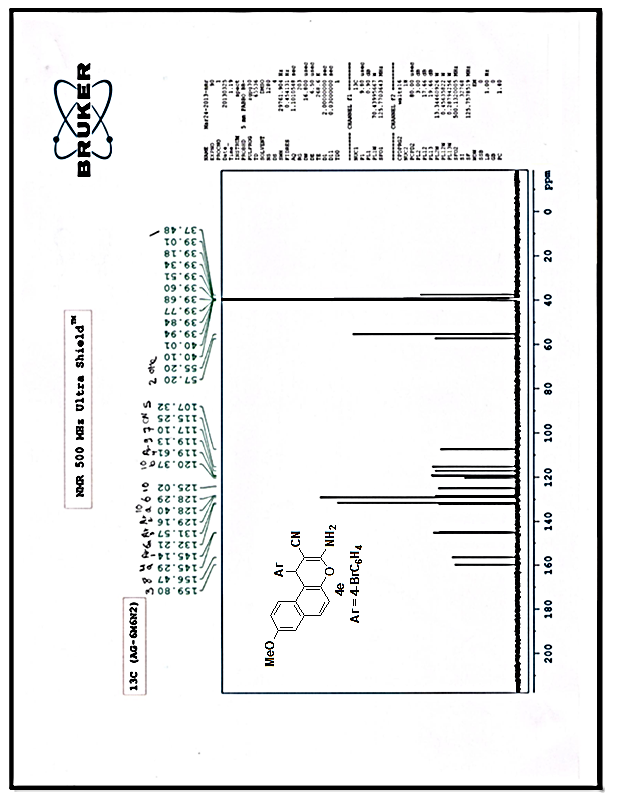


**Fig. S15.** ^13^C-NMR spectrum (DMSO-*d_6_*, 125 MHz) of compound **4e.**


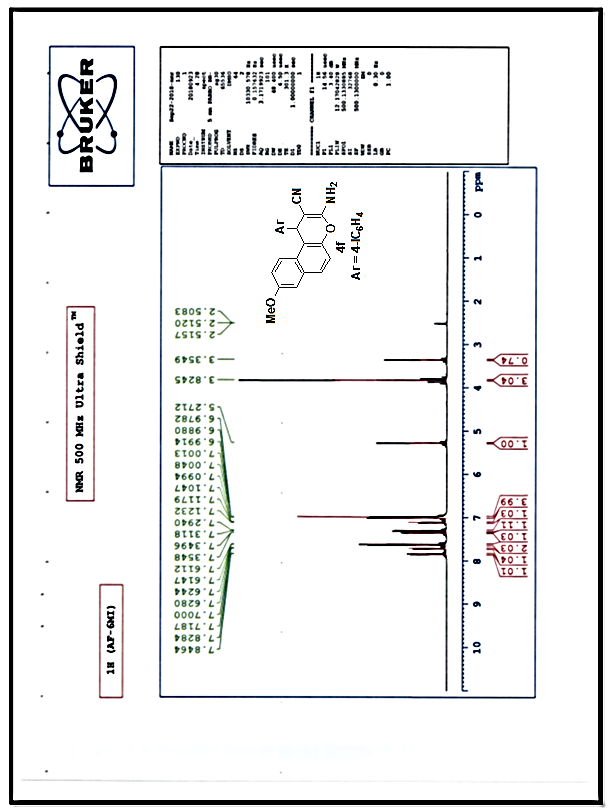


**Fig. S16.** ^1^H-NMR spectrum (DMSO-*d_6_*, 500 MHz) of compound **4f.**


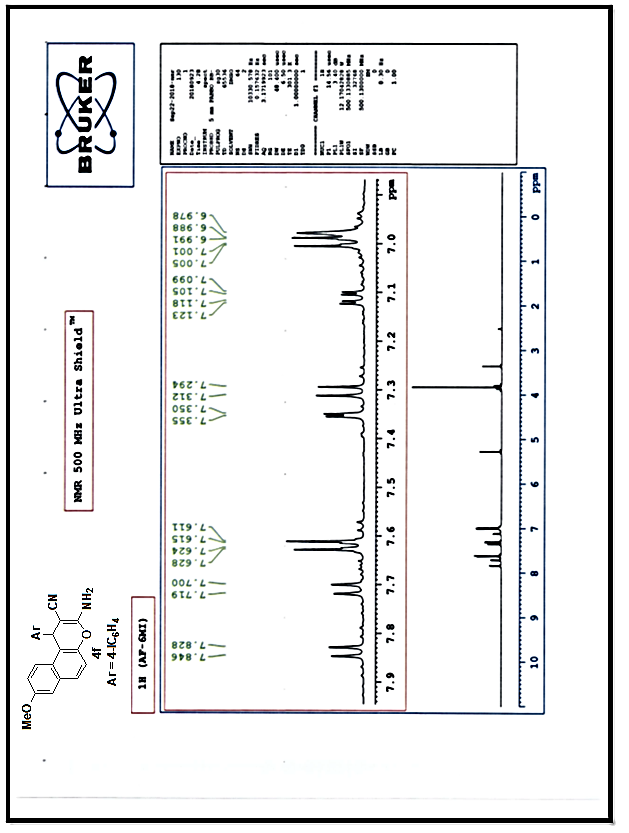


**Fig. S17.** Enlarged ^1^H-NMR spectrum (DMSO-*d_6_*, 500 MHz) of compound **4f.**


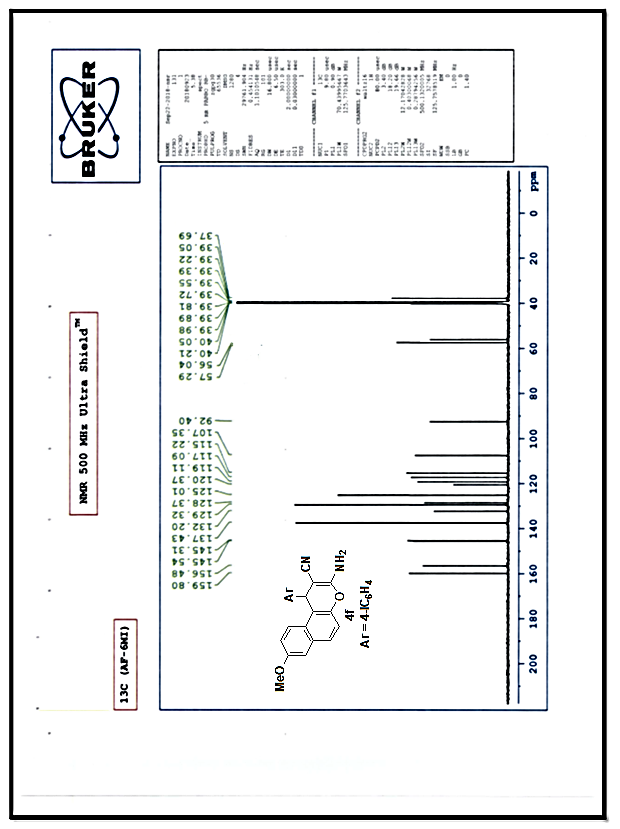


**Fig. S18.** ^13^C-NMR spectrum (DMSO-*d_6_*, 125 MHz) of compound **4f.**


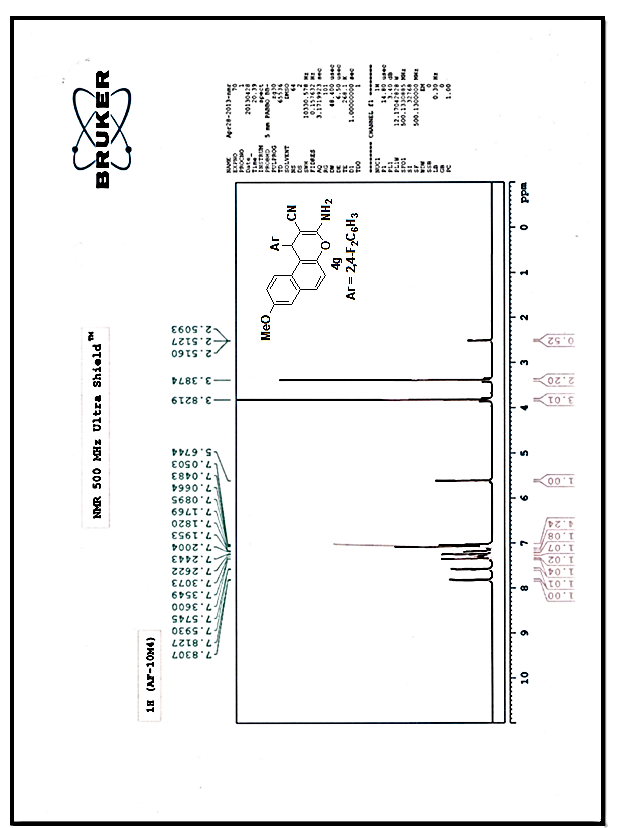


**Fig. S19**. ^1^H-NMR spectrum (DMSO-*d_6_*, 500 MHz) of compound **4g.**


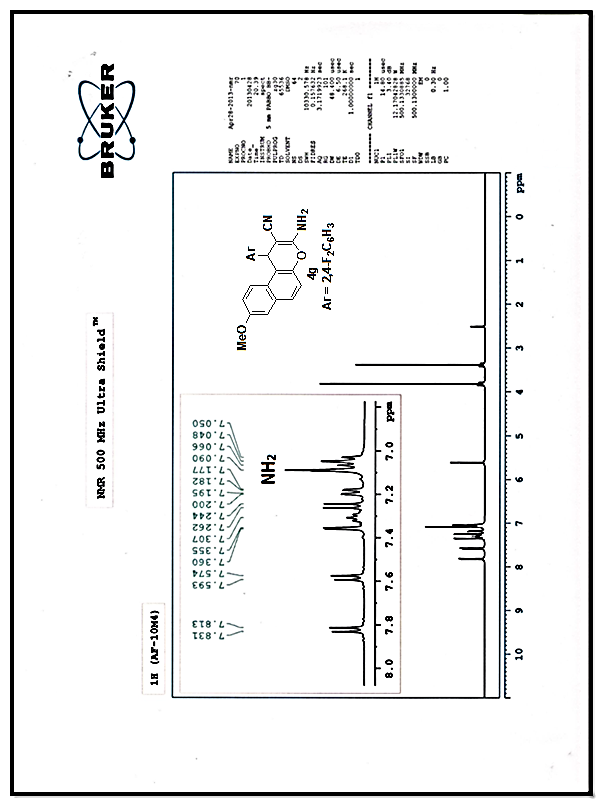


**Fig. S20**. Enlarged ^1^H-NMR spectrum (DMSO-*d_6_*, 500 MHz) of compound **4g.**


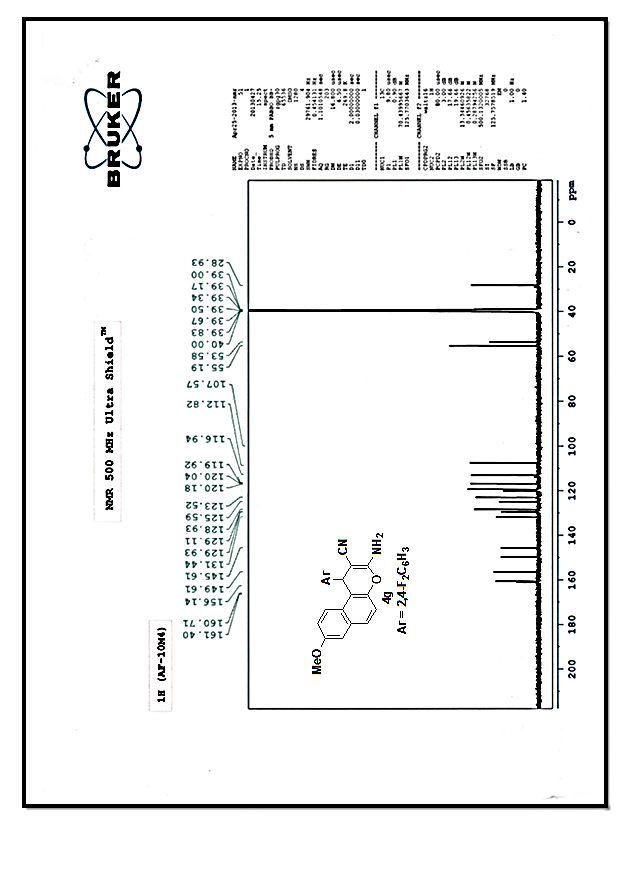


**Fig. S21.** ^13^C-NMR spectrum (DMSO-*d_6_*, 125 MHz) of compound **4g.**


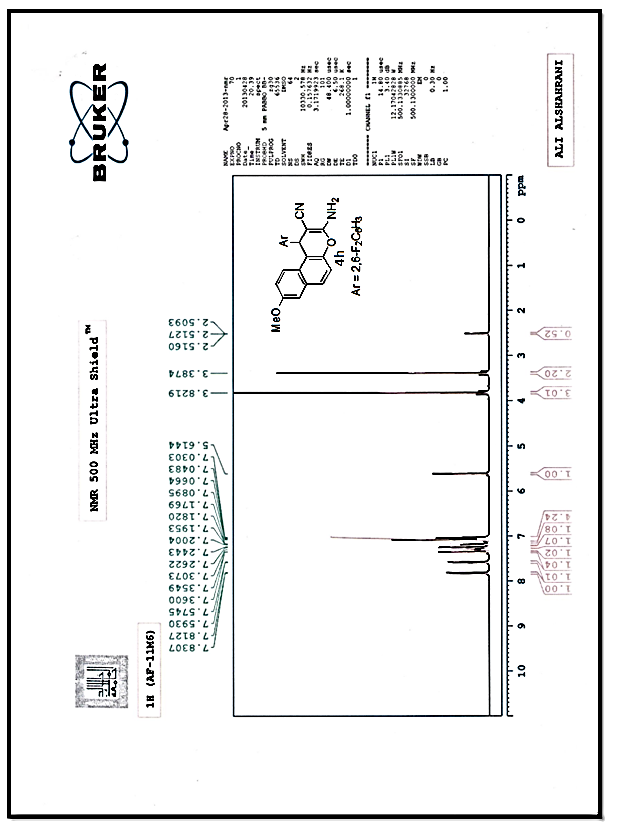


**Fig. S22.** ^1^H-NMR spectrum (DMSO-*d_6_*, 500 MHz) of compound **4h.**


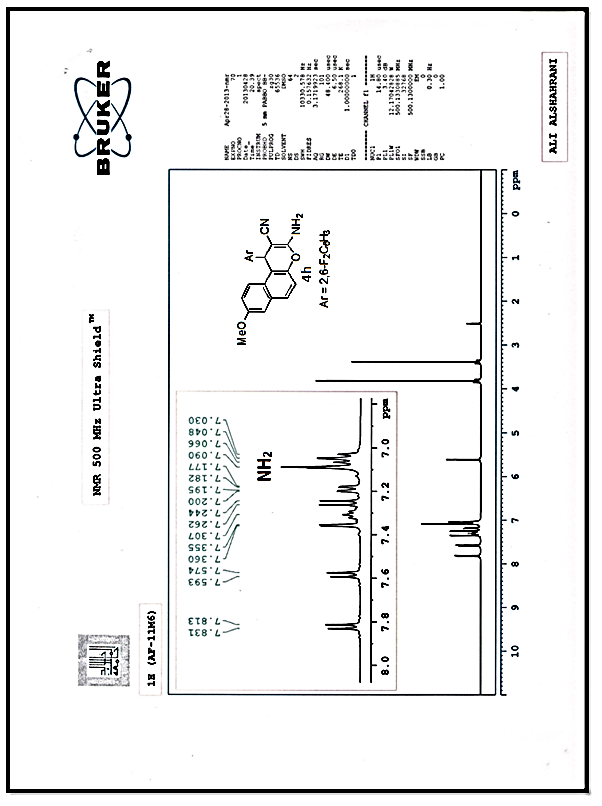


**Fig. S23.** Enlarged ^1^H-NMR spectrum (DMSO-*d_6_*, 500 MHz) of compound **4h.**


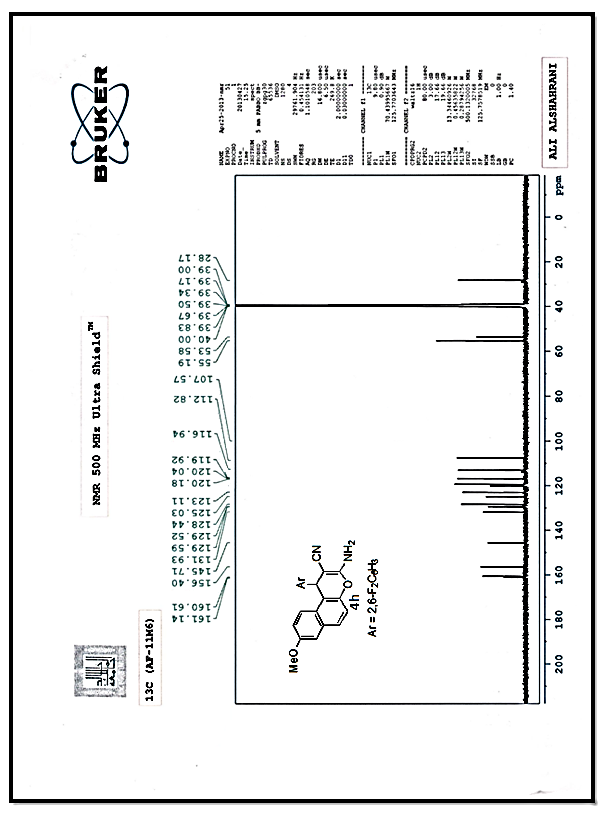


**Fig. S24.** ^13^C-NMR spectrum (DMSO-*d_6_*, 125 MHz) of compound **4h.**


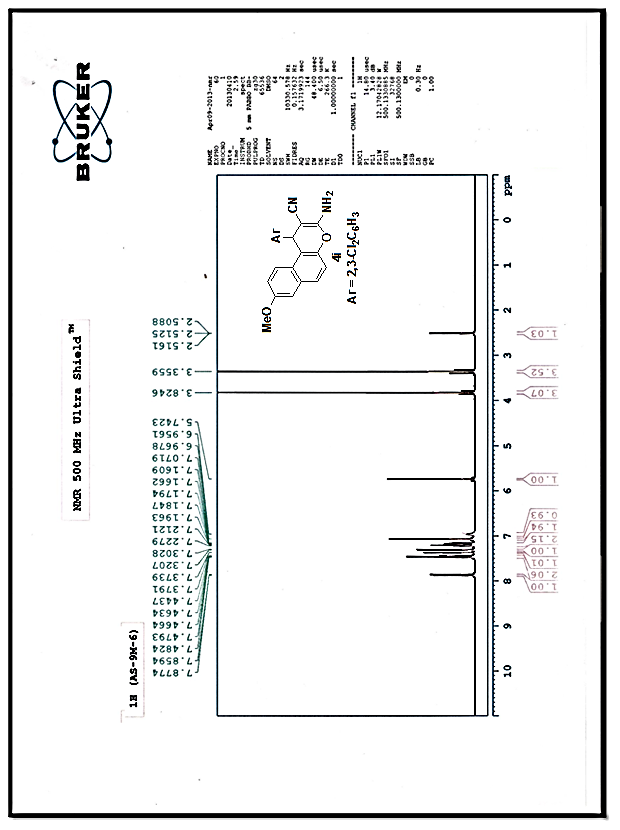


**Fig. S25.** ^1^H-NMR spectrum (DMSO-*d_6_*, 500 MHz) of compound **4i.**


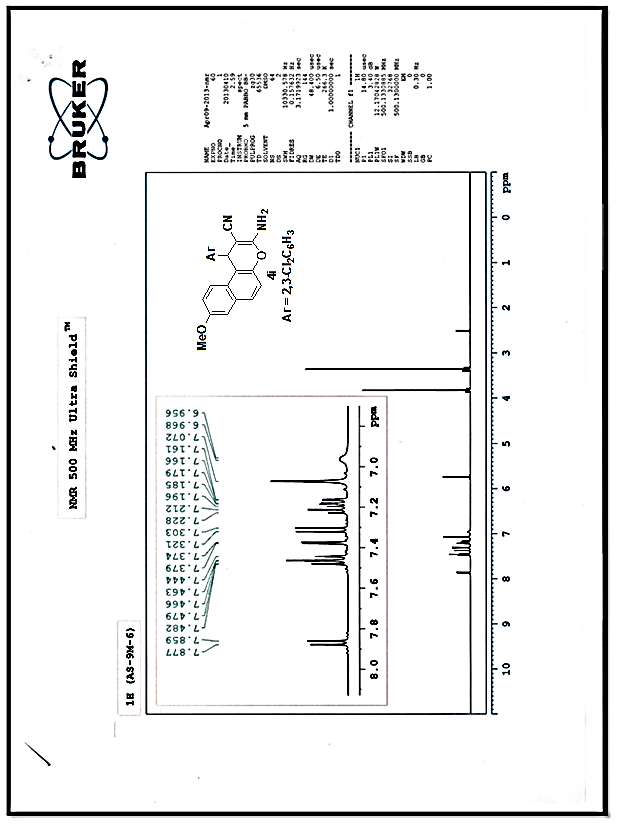


**Fig. S26.** Enlarged ^1^H-NMR spectrum (DMSO-*d_6_*, 500 MHz) of compound **4i.**


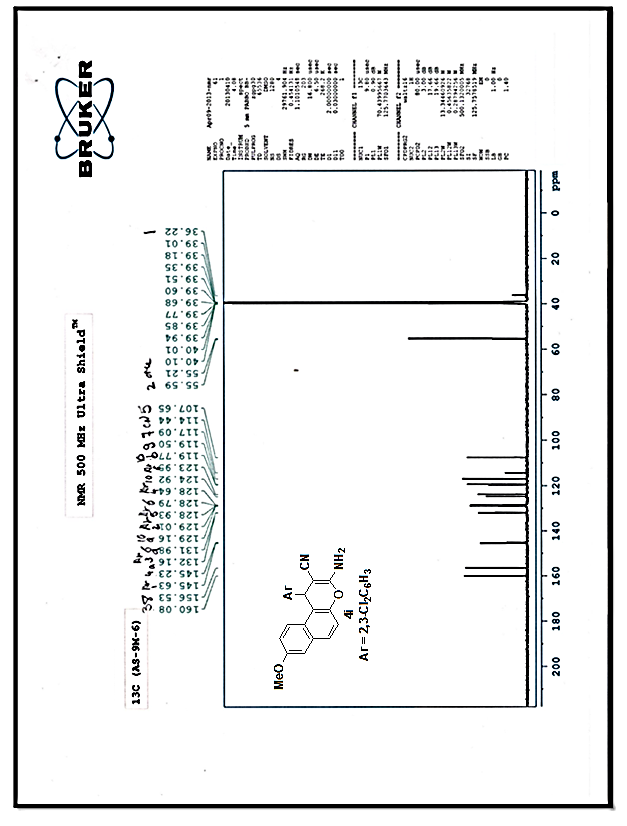


**Fig. S27.** 13C-NMR spectrum (DMSO-*d_6_*, 125 MHz) of compound **4i.**


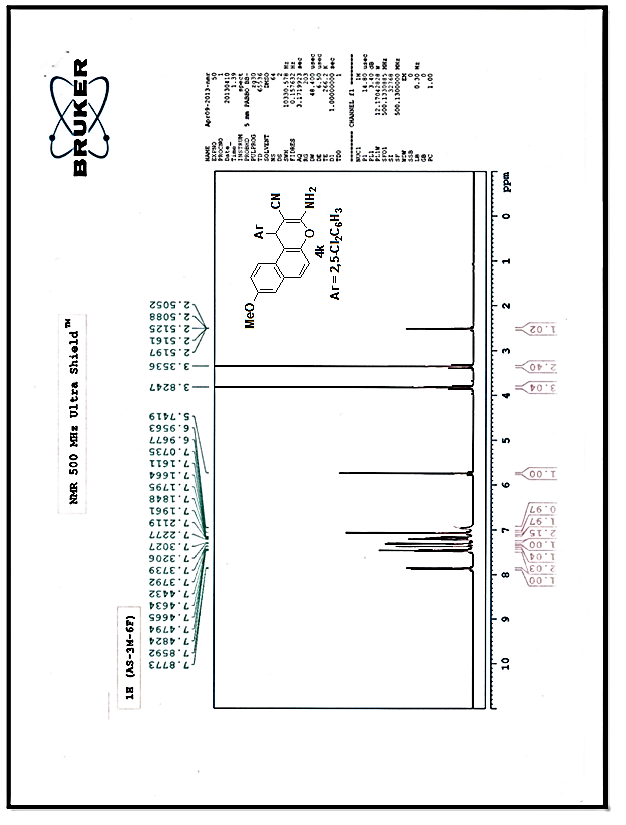


**Fig. S28.** ^1^H-NMR spectrum (DMSO-*d_6_*, 500 MHz) of compound **4k.**


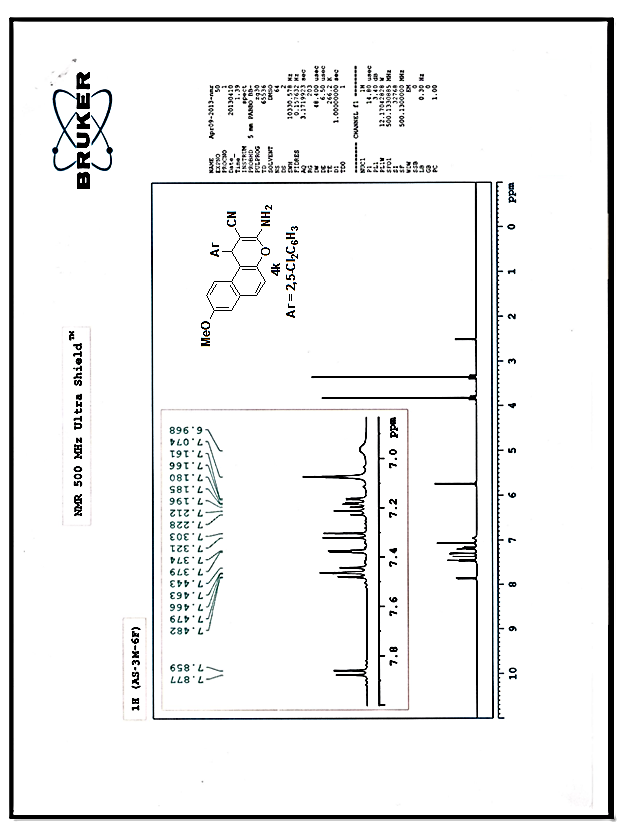


**Fig. S29.** Enlarged ^1^H-NMR spectrum (DMSO-*d_6_*, 500 MHz) of compound **4k.**


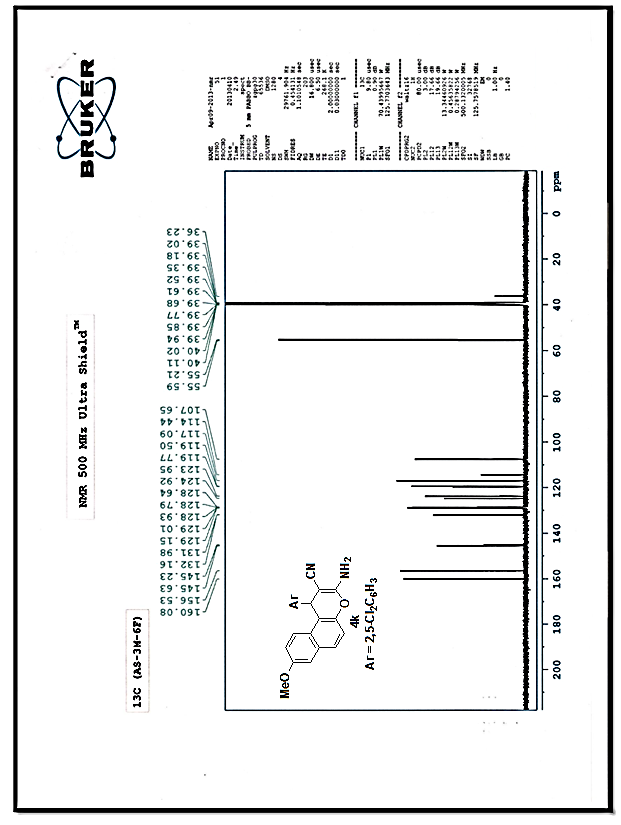


**Fig. S30.** ^13^C-NMR spectrum (DMSO-*d_6_*, 125 MHz) of compound **4k.**


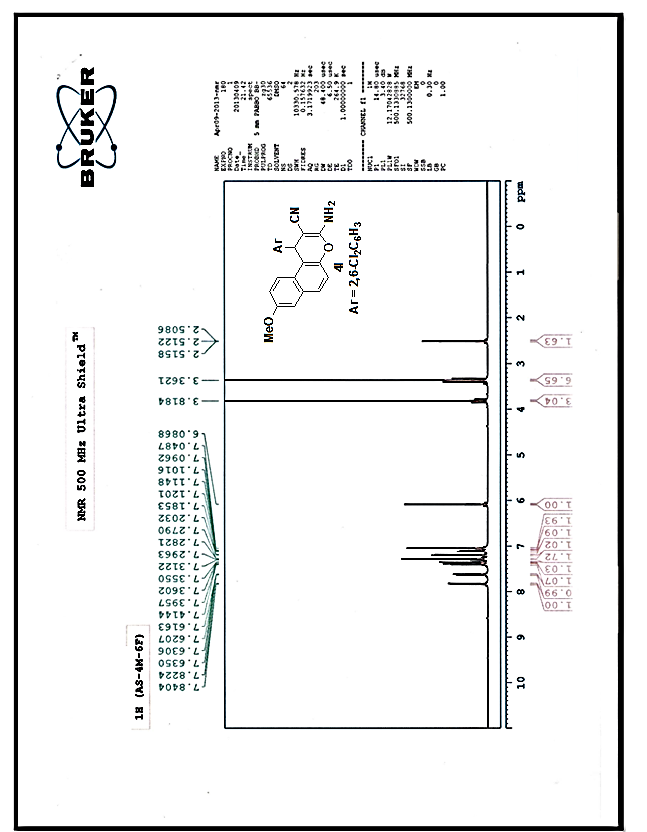


**Fig. S31.** ^1^H-NMR spectrum (DMSO-*d_6_*, 500 MHz) of compound **4l.**


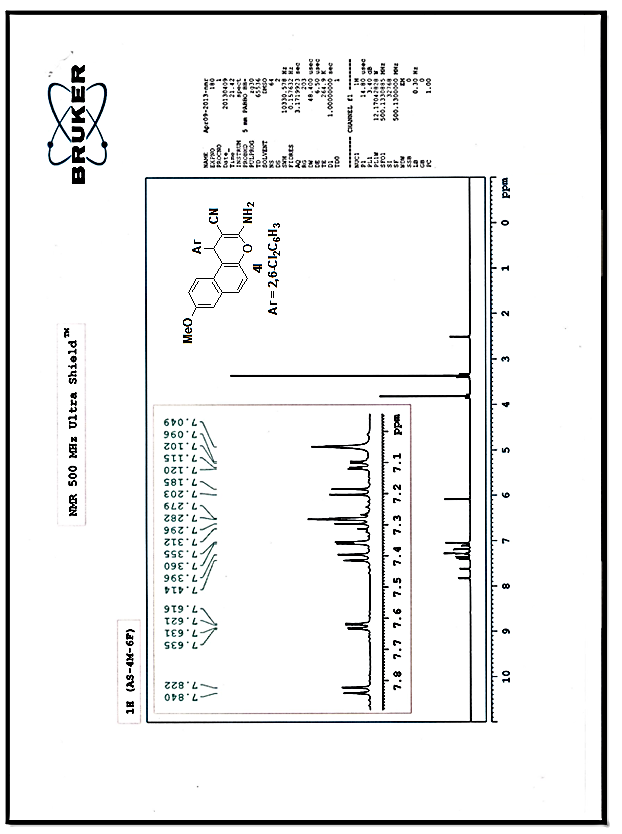


**Fig. S32.** Enlarged ^1^H-NMR spectrum (DMSO-*d_6_*, 500 MHz) of compound **4l.**


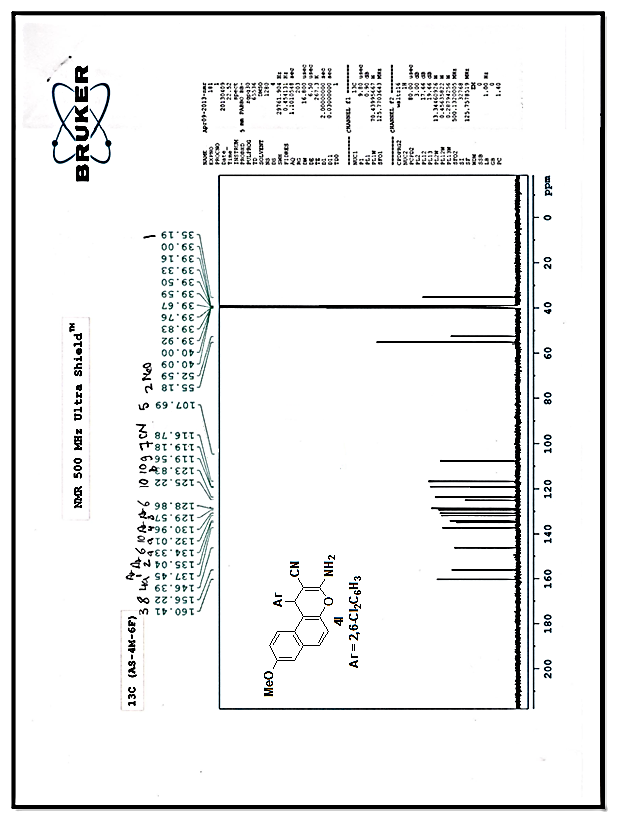


**Fig. S33**. ^13^C-NMR spectrum (DMSO-*d_6_*, 125 MHz) of compound **4l.**


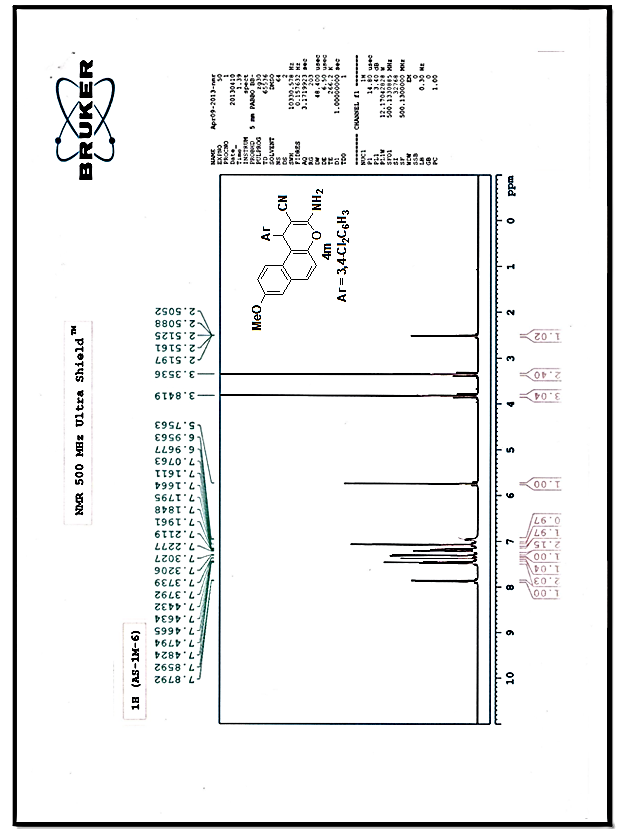


**Fig. S34.** ^1^H-NMR spectrum (DMSO-*d_6_*, 500 MHz) of compound **4m.**


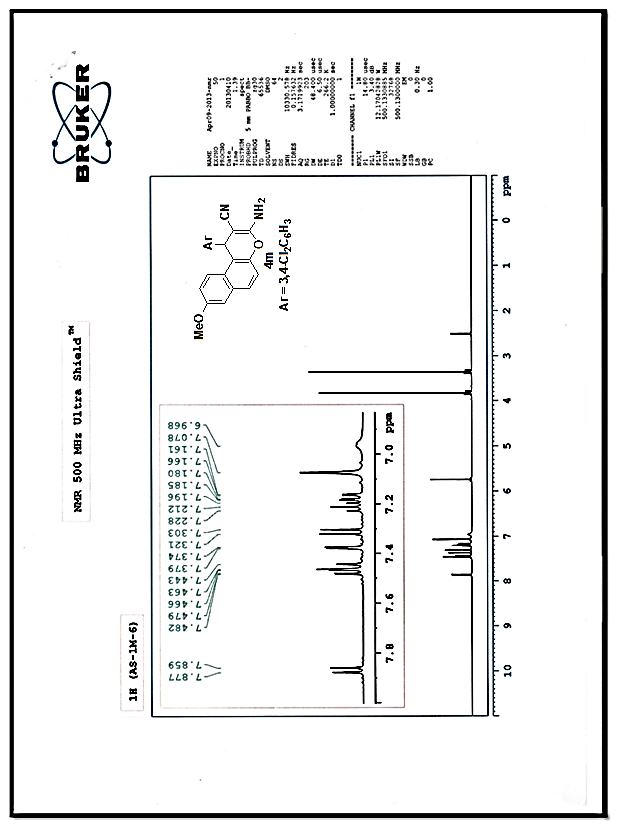


**Fig. S35.** Enlarged ^1^H-NMR spectrum (DMSO-*d_6_*, 500 MHz) of compound **4m.**


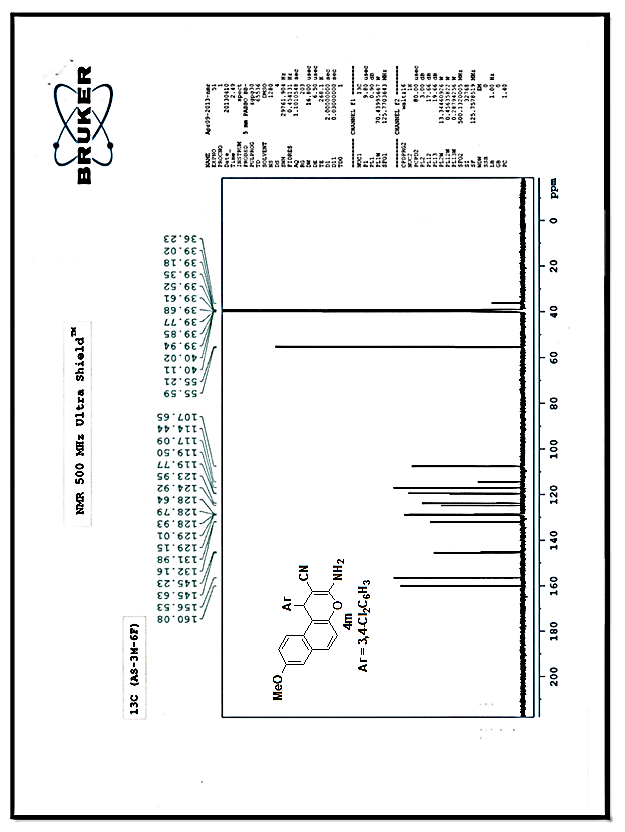


**Fig. S36.** ^13^C-NMR spectrum (DMSO-*d_6_*, 125 MHz) of compound **4m.**


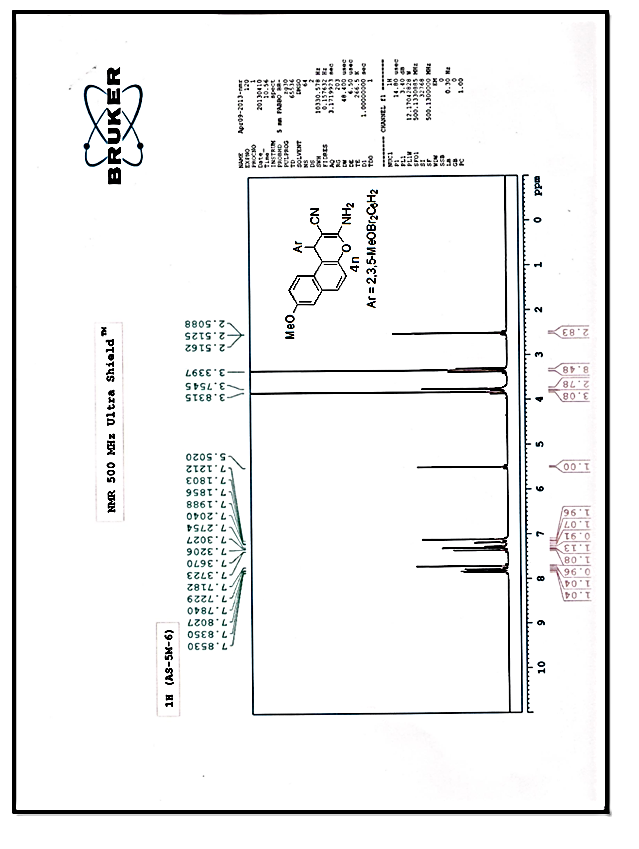


**Fig. S37.** ^1^H-NMR spectrum (DMSO-*d_6_*, 500 MHz) of compound **4n.**


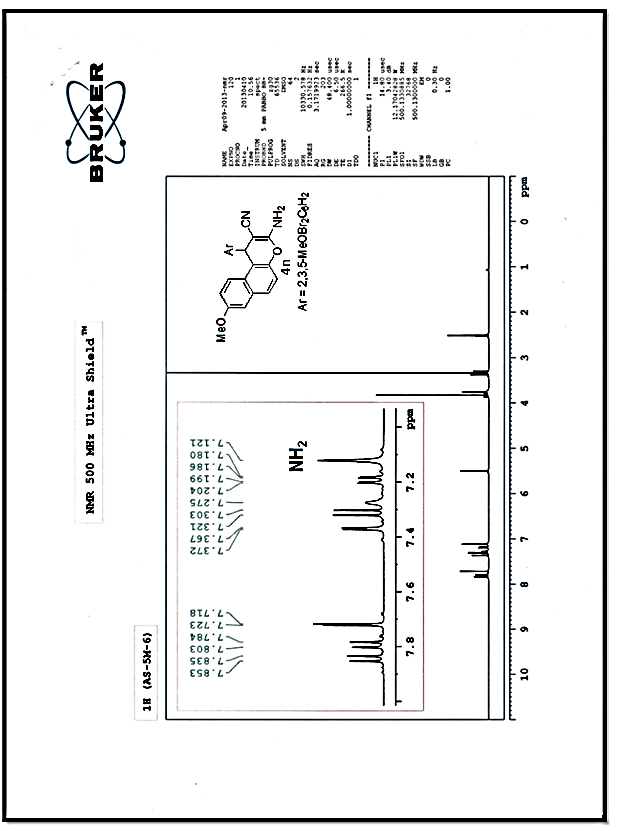


**Fig. S38**. Enlarged ^1^H-NMR spectrum (DMSO-*d_6_*, 500 MHz) of compound **4n.**


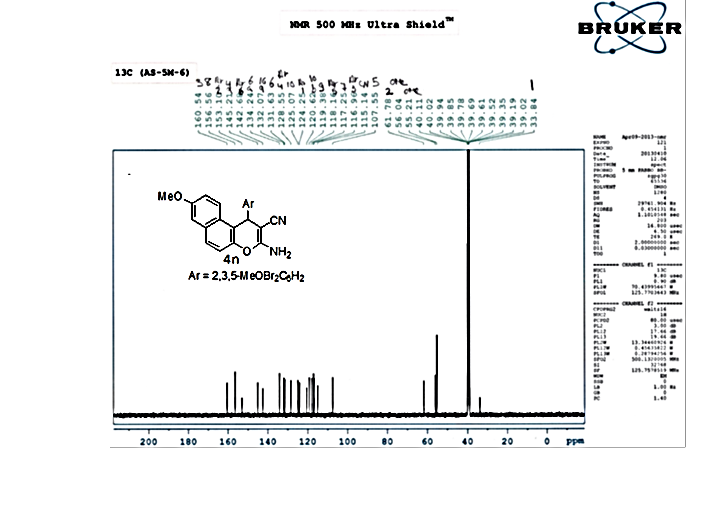


**Fig. S39.** ^13^C-NMR spectrum (DMSO-*d_6_*, 125 MHz) of compound **4n.**


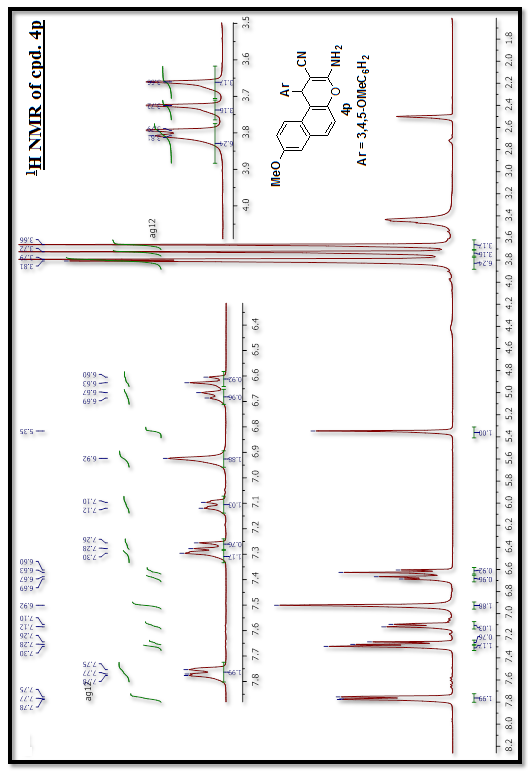


**Fig. S40.** ^1^H-NMR spectrum (DMSO-*d_6_*, 500 MHz) of compound **4p.**


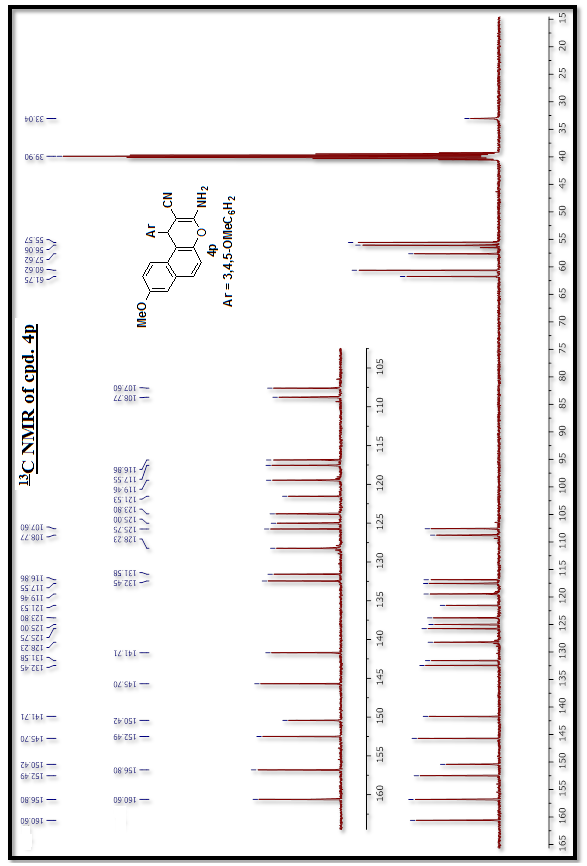


**Fig. S41.** ^13^C-NMR spectrum (DMSO-*d_6_*, 125 MHz) of compound **4p.**


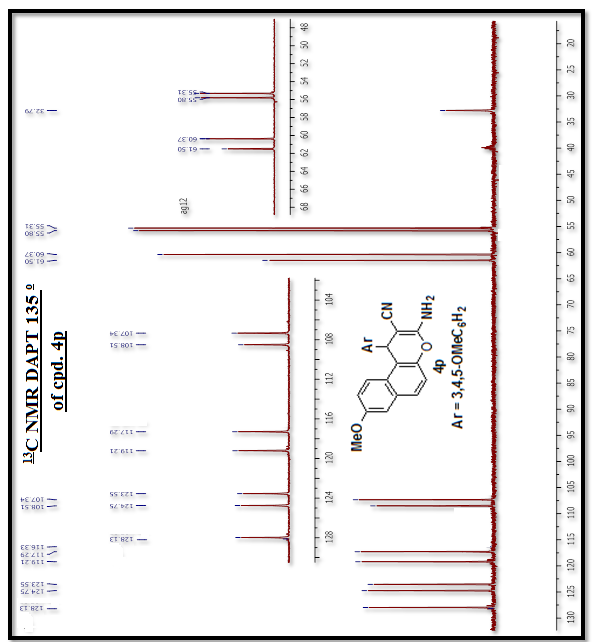


**Fig. S42.** DEPT spectrum (DMSO-*d_6_*, 125 MHz) of compound **4p.**


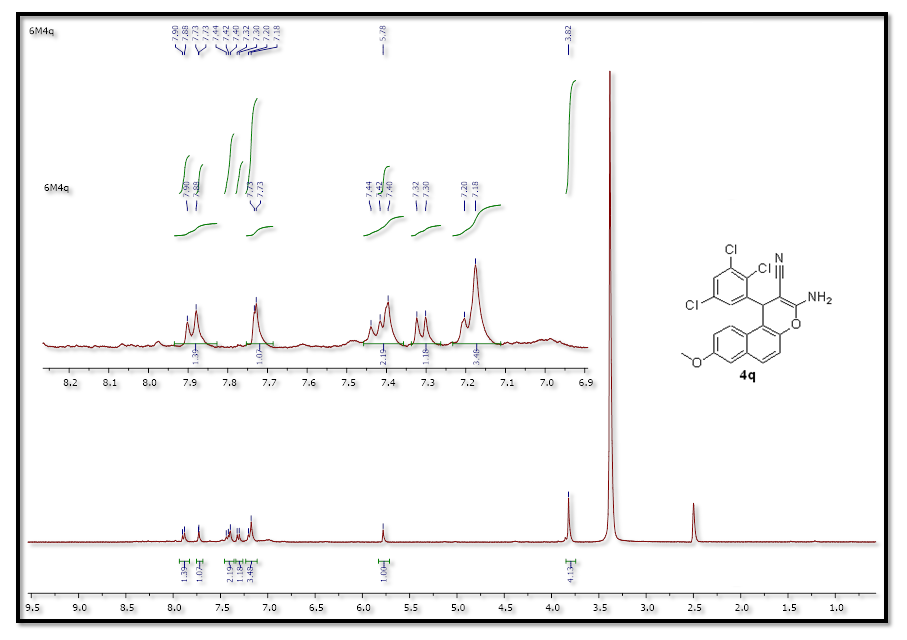


**Fig. S43.** ^1^H-NMR spectrum (DMSO-*d_6_*, 500 MHz) of compound **4q.**


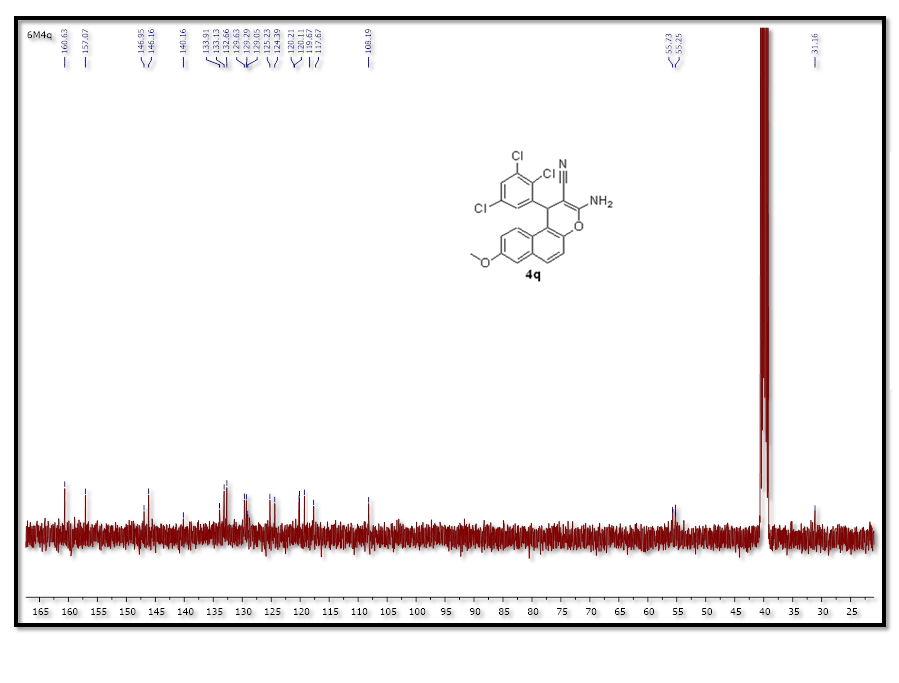


**Fig. S44**. ^13^C-NMR spectrum (DMSO-*d_6_*, 125 MHz) of compound **4q.**


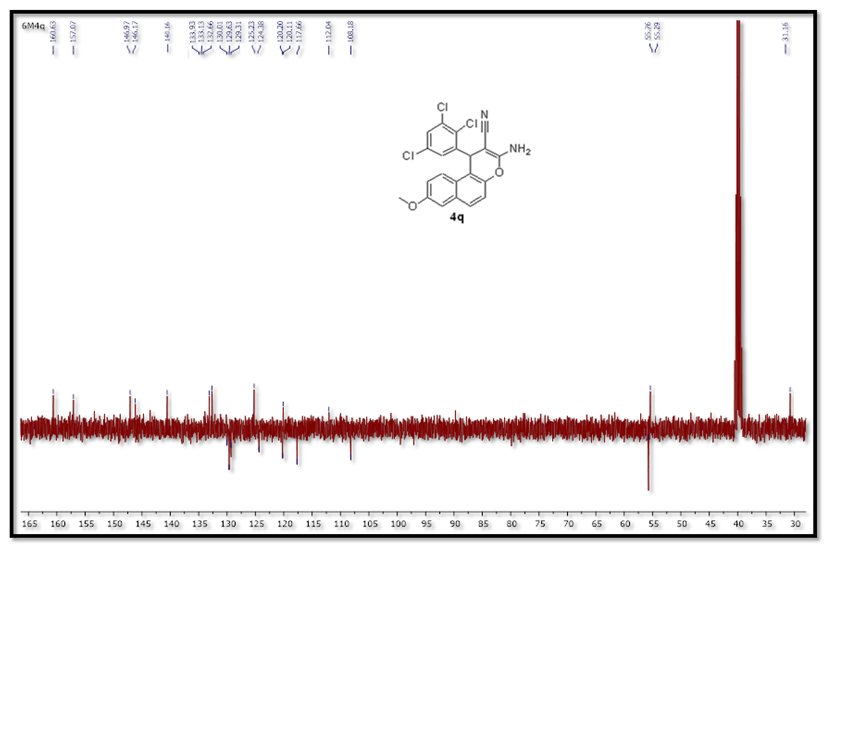


**Fig. S45.** APT spectrum (DMSO-*d_6_*, 125 MHz) of compound **4q.**
